# Human PZP and common marmoset A2ML1 as pregnancy related proteins

**DOI:** 10.1038/s41598-020-61714-8

**Published:** 2020-03-20

**Authors:** Hirofumi Kashiwagi, Hitoshi Ishimoto, Sun-ichiro Izumi, Toshiro Seki, Rihito Kinami, Asako Otomo, Kazumi Takahashi, Fuyuki Kametani, Noriaki Hirayama, Erika Sasaki, Takashi Shiina, Kou Sakabe, Mikio Mikami, Yoshie Kametani

**Affiliations:** 1 Department of Obstetrics and Gynecology, Tokai University School of Medicine, Isehara, Kanagawa Japan; 2 Department of Internal Medicine, Division of Nephrology, Endocrinology and Metabolism, Tokai University School of Medicine, Isehara, Kanagawa Japan; 3 Department of Molecular Life Science, Division of Basic Medical Science and Molecular Medicine, Tokai University School of Medicine, Isehara, Kanagawa Japan; 4 Department of Dementia and Higher Brain Function, Tokyo Metropolitan Institute of Medical Science, Setagaya, Tokyo Japan; 5 Division of Pharmaceutical Sciences, Institute of Advanced Biosciences, Tokai University School of Medicine, Isehara, Kanagawa Japan; 6 Central Institute for Experimental Animals, Kawasaki, Kanagawa Japan; 7 RIKEN Cluster for Pioneering Research, Wako, Saitama Japan; 8 Department of Anatomy, Division of Basic Medical Science, Tokai University School of Medicine, Isehara, Kanagawa Japan

**Keywords:** Evolutionary developmental biology, Zoology

## Abstract

While pregnancy-related proteins (PRP) are known to contribute to immunotolerance during pregnancy, their significance to development of invasive placenta is unclear. We compared PRP expression in humans and the common marmoset (*Callithrix jacchus*), a new-world monkey. Invasive placenta was observed at the maternal-foetal interface of marmoset placenta from green fluorescent protein (GFP)-expressing foetus and wild type mother. The pregnancy zone protein (PZP) and alpha-2 macroglobulin-like 1 (A2ML1) proteins exhibited the most prominent increase in expression during the second trimester in humans and marmoset, respectively. In humans, PZP accumulated at the maternal-foetal interface and A2ML1 accumulated in the amnion. Similarly, A2ML1 mRNA was detected in marmoset placenta. These proteins belong to the A2M family of protease inhibitors, and both PZP and A2ML1 share around 90% homology between human and marmoset and have highly conserved structures. However, the protease-reacting bait regions of the proteins had lower homology (56.8–60.7% in proteins) relative to the rest of the sequence. Notably, the cleavage site of a proinflammatory proline-endopeptidase was preserved in human PZP and marmoset A2ML1. These proteins contain multiple sites that are cleaved by proteases involving proline-endopeptidase. Systemic regulation of these A2M family proteins may be important in animals with invasive placenta.

## Introduction

The placental structure and molecular mechanism of pregnancy have been greatly changed during the evolution of eutherian mammals, even within the Euarchontoglires. For example, the rodent placenta develops a labyrinth-like structure with shallow interstitial invasion of trophoblasts, while the human placenta, which is evolutionarily newer than that of the macaque, is composed of trophoblasts that form chorions and deeply invade into the decidua to remodel maternal blood vessels^1,2^. In primates and rodents, trophoblast cells invading into the decidua construct a maternal-foetal interface, where semi-allograft foetal tissues are checked by the mother’s immunity. Primates have longer gestation periods and more highly invasive maternal-foetal interfaces than those of rodents^3^. As the placenta becomes more invasive, the amount of oxygen and nutrient transportation increases owing to the expansion of blood vessels and reduction of vascular resistance. In humans, it is reported that placental invasion is related to brain growth, as large amounts of oxygen and nutrients may develop larger brains^4,5^. However, this benefit forces the trophoblasts to encounter the mother’s immunity for a long period.

Furthermore, it has also been suggested that foetal trophoblast cells, tissue pieces, and foetal non-methylated CpG DNA are circulating systemically throughout the body of species with highly invasive trophoblasts that are retained for a long period^6^. As these systemically circulating foetal tissues and DNAs may cause allogeneic reactions or innate inflammatory reactions outside of the placenta, immunoregulation outside of the uterus is critical to the maintenance of the mother’s health^7^.

Pregnancy-related proteins (PRPs) are molecules whose concentration is increased in maternal circulation during pregnancy^8–10^. The functions of these proteins are not clearly defined, but it has been speculated that they help to maintain a normal pregnancy by suppressing immune reactions to allogeneic cells or fragments. Several molecules have been identified as PRPs, and their functions are being gradually elucidated^10,11^. For example, pregnancy specific beta-1-glycoprotein (PSG) contributes to the maternal immune system change from the inflammatory Th1 environment to an anti-inflammatory Th2 environment as well as to macrophage activity^9,12,13^. Contrastingly, human leucocyte antigen-G (HLA-G) is known to stimulate Th2 response and Treg activation upon chronic inflammation and viral infection, causing systemic immunosuppression, and induces immune tolerance at the maternal-foetal interface of the placenta^14–16^. Similarly, pregnancy zone protein (PZP) has been reported to be markedly increased in the maternal sera during pregnancy^17^, and binds to a molecule closely related to immunosuppression, transforming growth factor-β (TGF-β)^18,19^.

However, most functional analyses of PRPs have been performed using patient specimens or experimental animals, mainly murine systems^20–22^. Unlike experimental animals, voluntary specimens obtained in the hospital are sometimes from abnormal pregnancies, and do not come from the same temperature, food, and treatment conditions. Compared to experimental animals, which are usually maintained in a closed colony, human beings have highly diverged genetic backgrounds. Moreover, mice do not have a highly invasive placenta and have only a three-week gestation, so it is difficult to completely elucidate the role of PRPs in primates, which have invasive placentae and longer gestation, using mouse models. Therefore, model systems should be selected and/or developed based on animals with invasive trophoblasts and immune systems more similar to those of humans, which would be more convincing models of human pregnancy.

The common marmoset (*Callithrix jacchus*) is a new-world monkey with a five-month gestation, which is longer than that of rodents, and has many benefits as a human pregnancy model compared to other species^23–25^. Firstly, invasive trophoblasts have not been observed in prosimians but are present in placentae of new-world monkeys, although the structure of these invasive trophoblasts is a little different from that of humans, as their villi are connected by bridges of trophoblasts resulting in a trabecular condition^26^. Secondly, our group previously analysed the immune function of the marmoset and described the similarities and differences between human and marmoset immunity^25^. As the nucleotide homology of 30 immune-related genes between human and marmoset is about 86%, marmoset immunity is considered to be evolutionally equidistant from humans and mice^27^. Thirdly, gene modification is possible in the marmoset and transgenic animals are already available, as we have previously reported green fluorescent protein (GFP)-expressing marmosets^23^. Moreover, additional gene editing techniques are also being explored^24^.

Contrastingly, marmosets, which often conceive multiple twins, have the randomly attaching part of the umbilical cord in the placenta divided into two lobes, and the umbilical cord blood vessels are connected among foetuses in the placenta^28,29^. As a result, the blood of foetuses mixes and encounters allogeneic tissues. Therefore, the marmoset pregnancy system requires stronger immunosuppressive function than that of human, whose blood never mixes in the placenta of twins.

In this study, we performed a comparative analysis of PRPs fluctuating during pregnancy in both the plasma and placenta of marmosets and humans to clarify the role of PRPs shared by these two species.

## Results

### Histochemical comparison of human and marmoset placenta

**Figure 1:**
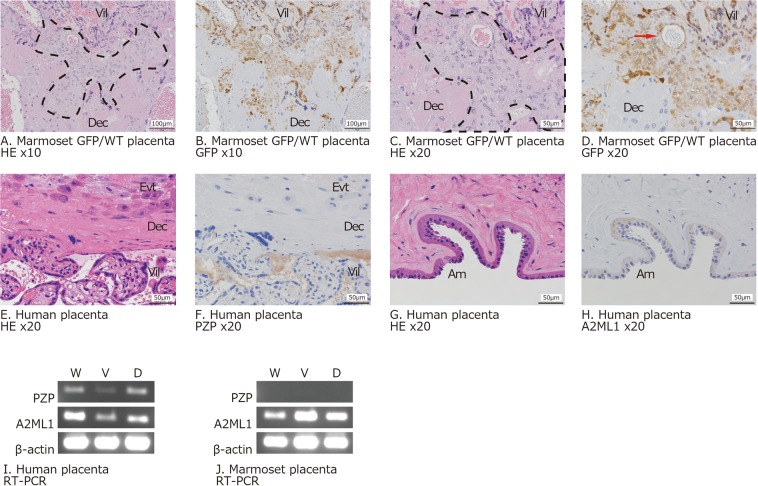
Histochemical analysis of human and marmoset placenta. Panels A–D: Infiltration of foetal trophoblasts in marmoset placenta detected by immunohistochemistry. GFP/WT indicates green fluorescent protein foetus/wild type mother. Low (**A**) and high (**C**) power field images of haematoxylin-eosin (HE)-stained villi-decidua interface of representative GFP marmoset placenta. Low (**B**) and high (**D**) power field images of GFP expression detected by anti-GFP MAB and DAB. Brown signals (DAB) show GFP-positive cells. Area surrounded by dashed line shows invading cell mass with large nuclei in the large cytoplasm. Vil: villi; Dec: decidua; Evt: extra villous trophoblast; Am: amnion; red arrows: blood vessels in the maternal-foetal interface. Panels E–H: Immunohistochemical analysis of PZP and A2ML1 localisation in representative human placenta (No. H6). HE staining of human term placenta (anchoring villi) (**E**). PZP expression detected by anti-PZP antibody (**F**). HE staining of human term placenta (amnion) (**G**). A2ML1 expression detected by anti-A2ML1 antibody (**H**). Brown signals (DAB) show PZP/A2ML1-positive cells. Scale bars indicate 100 (**A**,**B**) and 50 µm (**C**–**H**). Panels I and J: Tissue-specific expression of PZP and A2ML1 mRNA detected by semi-quantitative RT-PCR. Representative samples (No. H6 and No. M7) were shown. All the samples analysed in this study are shown in Supplementary Fig. 2. W: whole placenta; V: villi; D: decidua; β-actin: positive control.

We analysed the structure of full-term marmoset placenta and examined the extent of trophoblast invasion. Extravillous trophoblasts were observed in the decidua (Fig. 1A–D). In marmoset placenta, villi with trabecular condition and narrow intervillous space were observed (Fig. 1A,C) as reported previously^26^. A unique cell mass with large nuclei in the large cytoplasm was found around the decidua near the adherent part of the villi. These cells infiltrated into the decidua and were distinctive. Collectively, haematoxylin-eosin (HE) staining showed that the villi of the marmoset placenta invaded into the maternal decidua as did those of the human placenta.

To confirm infiltration of foetal trophoblasts into the decidua, the placenta from a GFP foetus-wild type (WT) mother was obtained and tissue sections were examined. Representative data of three GFP marmoset specimens are shown in Fig. 1B,D. GFP expression was detected by immunohistochemistry (IHC), revealing GFP-positive foetal cells infiltrating into GFP-negative decidua and involving blood vessels in the maternal-foetal interface. The cells were deeply dispersed in the decidua, although the depth of the marmoset’s decidua is shorter than that in human placenta. These results suggested that marmoset placenta is simply invasive, similar to that of old-world monkeys and humans.

### Comparison of PRPs in human and marmoset plasma

Because the invasion of marmoset trophoblasts into the decidua was similar to that in humans, and the period of pregnancy was almost five months, we hypothesized that systemic immune regulation is necessary for marmoset pregnancy. Therefore, we used liquid chromatography-mass spectrometry (LC-MS) to obtain a profile of plasma protein variation during pregnancy in humans and marmosets.

A total of 83 and 69 proteins were identified in placentae of human and marmoset, respectively (Supplemental Table 1). These proteins were classified into three groups (increased, decreased, and no-change proteins during pregnancy), and proteins which showed changes (described in Table 1 legend) during pregnancy are listed in Table 1. In humans, the PRPs PZP, PSG1, and PSG9 increased during pregnancy, although the difference was not statistically significant. A2ML1 was significantly increased in marmosets during pregnancy but not in humans, indicating that it acts as a PRP in marmoset but not in humans. Meanwhile, PZP was not increased in marmoset, suggesting that it is not a PRP in marmoset. PSG proteins are another well-known PRP family in humans. Although a PSG-8-like gene was recognised as a PSG homolog in the marmoset genome (assembly callithrix jacchus-3.2), its expression has not been reported and it was not detected in the current study. The score plot of principal component analysis (PCA) showed two distinct clusters, i.e. pregnant group and non-pregnant group. The PCA loading plot revealed that marmoset A2ML1 protein (gi | 6756777) was furthermost located (Supplemental Fig. 1F). Therefore, A2ML1 was considered to be the most important molecule in marmoset during pregnancy.

**Table 1:** Human (A) and marmoset (B) serum proteins with significant changes during pregnancy.

A. Human																								
Categry		No.	Identified Proteins	Pregnancy related proteins (P.R.P)	Protease Inhibitors	UniProt EntryName	UniProt Accession No.	Molecular Weight	Average			ratio		ratio		1st trimester			2nd trimester			3rd trimester		
					(P.I.)/Proteases				1st	2nd	3rd	2nd/1st	*p*	3rd/1st	*p*	H1	H2	H3	H1	H2	H3	H1	H2	H3
1	1a	1	Pregnancy zone protein	P.R.P.	P.I.	PZP_HUMAN	P20742	164 kDa	2.77	11.32	7.25	4.08	0.09	2.61	0.21	8.32	0.00	0.00	25.59	4.32	4.03	21.75	0.00	0.00
		2	Antithrombin-III		P.I.	ANT3_HUMAN	P01008	53 kDa	0.33	2.02	0.31	6.07	0.26	0.92	0.21	0.00	0.00	1.00	0.00	6.05	0.00	0.00	0.00	0.92
		3	Alpha-1-antichymotrypsin		P.I.	AACT_HUMAN	P01011	48 kDa	0.00	2.02	0.00		0.21			0.00	0.00	0.00	0.00	6.05	0.00	0.00	0.00	0.00
		4	Complement C1q subcomponent subunit C			C1QC_HUMAN	P02747	26 kDa	0.00	1.15	0.00		0.21			0.00	0.00	0.00	0.00	3.46	0.00	0.00	0.00	0.00
	1b	5	Serotransferrin			TRFE_HUMAN	P02787	77 kDa	34.12	52.12	97.23	1.53	0.17	2.85	0.14	39.51	30.93	31.92	29.68	69.20	57.49	86.98	26.89	177.83
		6	Apolipoprotein C-II			APOC2_HUMAN	P02655	11 kDa	10.65	12.57	23.27	1.18	0.26	2.18	0.02	5.20	12.80	13.96	7.16	10.38	20.17	20.71	28.01	21.08
		7	Sex hormone-binding globulin			SHBG_HUMAN	P04278	44 kDa	9.52	15.90	23.72	1.67	0.01	2.49	0.02	13.52	1.07	13.96	20.47	6.05	21.18	28.99	20.17	22.00
		8	Fibronectin			FINC_HUMAN	P02751	263 kDa	4.82	6.10	15.77	1.27	0.40	3.27	0.03	0.00	7.47	6.98	0.00	17.30	1.01	15.53	13.45	18.33
		9	Apolipoprotein L1			APOL1_HUMAN	O14791	44 kDa	3.09	2.55	6.96	0.83	0.12	2.25	0.08	2.08	3.20	3.99	1.02	2.60	4.03	6.21	10.08	4.58
		10	Hemoglobin subunit beta			HBB_HUMAN	P68871	16 kDa	0.36	0.97	4.40	2.73	0.24	12.37	0.17	0.00	1.07	0.00	2.05	0.86	0.00	3.11	0.00	10.08
		11	Hemoglobin subunit alpha			HBA_HUMAN	P69905	15 kDa	0.00	1.65	3.75		0.07		0.15	0.00	0.00	0.00	3.07	0.86	1.01	2.07	0.00	9.17
		12	Fibulin-1			FBLN1_HUMAN	P23142	77 kDa	0.00	0.91	6.95		0.11		0.02	0.00	0.00	0.00	0.00	1.73	1.01	5.18	5.60	10.08
		13	Complement component C8 alpha chain			CO8A_HUMAN	P07357	65 kDa	0.00	0.29	1.02		0.21		0.002	0.00	0.00	0.00	0.00	0.86	0.00	1.04	1.12	0.92
		14	Pregnancy-specific beta-1-glycoprotein 1	P.R.P.		PSG1_HUMAN	P11464	47 kDa	0.00	0.00	18.32				0.02	0.00	0.00	0.00	0.00	0.00	0.00	21.75	11.20	22.00
		15	Actin, cytoplasmic 1			ACTB_HUMAN	P60709	42 kDa	0.00	0.00	7.33				0.21	0.00	0.00	0.00	0.00	0.00	0.00	0.00	0.00	22.00
		16	Pregnancy-specific beta-1-glycoprotein 9	P.R.P.		PSG9_HUMAN	Q00887	48 kDa	0.00	0.00	4.94				0.10	0.00	0.00	0.00	0.00	0.00	0.00	9.32	0.00	5.50
2	2a	17	Complement C5			CO5_HUMAN	P01031	188 kDa	10.65	4.28	5.49	0.40	0.04	0.52	0.04	8.32	10.67	12.97	1.02	1.73	10.09	5.18	6.72	4.58
		18	Complement C1s subcomponent			C1S_HUMAN	P09871	77 kDa	2.76	1.01	1.77	0.37	0.10	0.64	0.13	2.08	3.20	2.99	0.00	0.00	3.03	1.04	3.36	0.92
		19	Plasma kallikrein		Protease	KLKB1_HUMAN	P03952	71 kDa	2.35	0.96	1.29	0.41	0.20	0.55	0.22	0.00	1.07	5.98	0.00	0.86	2.02	0.00	1.12	2.75
		20	Carboxypeptidase N subunit 2		Protease	CPN2_HUMAN	P22792	61 kDa	1.34	0.00	1.53		0.13	1.14	0.42	1.04	0.00	2.99	0.00	0.00	0.00	0.00	0.00	4.58
	2b	21	Ig gamma-1 chain C region			IGHG1_HUMAN	P01857	36 kDa	24.73	20.00	10.54	0.81	0.89	0.43	0.02	29.12	18.13	26.93	22.52	23.36	14.12	9.32	6.72	15.58
		22	Ig lambda-2 chain C regions			LAC2_HUMAN	P0CG05	11 kDa	9.42	11.09	4.37	1.18	0.11	0.46	0.02	7.28	16.00	4.99	7.16	19.03	7.06	4.14	8.96	0.00
		23	Ig kappa chain C region			IGKC_HUMAN	P01834	12 kDa	2.76	3.14	1.00	1.14	0.32	0.36	0.10	2.08	3.20	2.99	3.07	4.32	2.02	2.07	0.00	0.92
		24	Histidine-rich glycoprotein			HRG_HUMAN	P04196	60 kDa	1.76	0.97	0.00	0.55	0.21		0.10	2.08	3.20	0.00	2.05	0.86	0.00	0.00	0.00	0.00
		25	Inter-alpha-trypsin inhibitor heavy chain H3		P.I.	ITIH3_HUMAN	Q06033	100 kDa	1.40	0.68	2.59	0.49	0.21	1.84	0.28	2.08	2.13	0.00	2.05	0.00	0.00	1.04	6.72	0.00
		26	Leucine-rich alpha-2-glycoprotein			A2GL_HUMAN	P02750	38 kDa	0.68	1.25	0.31	1.85	0.21	0.45	0.19	1.04	0.00	1.00	1.02	1.73	1.01	0.00	0.00	0.92
**B. Marmoset**																								
Catgory		No.	Identified Proteins	Pregnancy related proteins (P.R.P)	Protease Inhibitors	NCBI GI No.	Accession No.	Molecular Weight	Average			ratio		ratio		50 days			100 days			120 days		
					(P.I.)/Proteases				1st	2nd	3rd	2nd/1st	***p***	3rd/1st	***p***	M1	M2	M3	M1	M2	M3	M1	M2	
1	1a	1	Alpha-2-macroglobulin-like	P.R.P.	P.I.	675677766	XP_002752342	165 kDa	15.07	259.86	55.58	17.25	0.01	3.69		31.05	0.00	14.15	331.99	217.41	230.18	75.30	35.86	
		2	Hemoglobin alpha chain			122366	P18972*	15 kDa	9.41	128.91	27.39	13.69	0.001	2.91		10.09	9.06	9.10	120.17	138.98	127.57	7.44	47.34	
		3	Hemoglobin beta chain			122577	P18985*	16 kDa	4.72	74.62	20.26	15.79	0.01	4.29		2.33	6.79	5.05	72.83	58.57	92.44	4.65	35.86	
		4	Apolipoprotein A-II			296229454	XP_002760286	11 kDa	1.53	5.03	4.52	3.29	0.08	2.96		1.55	0.00	3.03	1.82	4.96	8.32	1.86	7.17	
		5	Complement component C6			390460019	XP_002745070	106 kDa	0.52	4.27	1.39	8.26	0.01	2.70		1.55	0.00	0.00	6.07	3.97	2.77	2.79	0.00	
		6	Pigment epithelium-derived factor			675657740	XP_008994618	47 kDa	0.97	4.63	0.93	4.76	0.25	0.96		0.78	1.13	1.01	0.00	13.90	0.00	1.86	0.00	
		7	Immunoglobulin heavy chain variable region			20502564	AAM22526**	15 kDa	0.52	1.99	0.46	3.85	0.13	0.90		1.55	0.00	0.00	1.21	1.99	2.77	0.93	0.00	
		8	Zinc-alpha-2-glycoprotein-like			675642469	XP_008981062	27 kDa	0.26	1.94	0.46	7.48	0.09	1.80		0.78	0.00	0.00	3.03	0.00	2.77	0.93	0.00	
		9	Insulin-like growth factor-binding protein complex acid labile subunit			296219288	XP_002755809	66 kDa	0.52	1.34	0.46	2.59	0.10	0.90		1.55	0.00	0.00	3.03	0.99	0.00	0.93	0.00	
		10	Apolipoprotein E			296234066	XP_002762273	36 kDa	0.00	1.73	0.00		0.09			0.00	0.00	0.00	2.43	0.00	2.77	0.00	0.00	
		11	Complement component C8 beta chain			296208073	XP_002750920	67 kDa	0.00	1.04	0.46		0.003			0.00	0.00	0.00	1.21	0.99	0.92	0.93	0.00	
	1b	12	Plasma serine protease inhibitor		P.I.	675692410	XP_002754293	50 kDa	0.93	2.25	2.87	2.41	0.22	3.08		0.78	0.00	2.02	0.00	3.97	2.77	0.00	5.74	
2	2a	13	Haptoglobin			296231486	XP_002761163	38 kDa	71.36	0.00	10.69	0.00	0.06	0.15		87.70	19.24	107.14	0.00	0.00	0.00	21.38	0.00	
	2b	14	Angiotensinogen			675750692	XP_008984059	54 kDa	29.15	22.65	11.49	0.78	0.13	0.39		32.60	21.51	33.35	17.60	19.86	30.51	15.80	7.17	
		15	Alpha-1-acid glycoprotein 1			296229919	XP_002760492	25 kDa	14.29	6.26	3.29	0.44	0.05	0.23		16.30	21.51	5.05	6.07	9.93	2.77	3.72	2.87	
		16	Complement C4-A			675651995	XP_002746418	193 kDa	14.26	14.11	4.65	0.99	0.03	0.33		20.96	5.66	16.17	12.14	8.93	21.26	9.30	0.00	
		17	Gelsolin			675639222	XP_009000537	86 kDa	8.00	4.69	3.04	0.59	0.08	0.38		7.76	10.19	6.06	5.46	3.97	4.62	4.65	1.43	
		18	Leucine-rich alpha-2-glycoprotein			296232579	XP_002761694	38 kDa	7.58	5.94	0.00	0.78	0.23			5.43	1.13	16.17	1.21	12.91	3.70	0.00	0.00	
		19	Complement factor H			675747009	XP_008983383	140 kDa	5.10	4.57	1.86	0.90	0.40	0.36		6.99	2.26	6.06	7.89	3.97	1.85	3.72	0.00	
		20	Serum amyloid A-4 protein			675698869	XP_009006030	15 kDa	4.63	2.46	1.39	0.53	0.12	0.30		5.43	3.40	5.05	1.82	0.00	5.55	2.79	0.00	
		21	Plasminogen		Protease	675655078	XP_008993588	15 kDa	3.62	5.60	0.00	1.55	0.24			10.87	0.00	0.00	10.32	0.00	6.47	0.00	0.00	
		22	Aminopeptidase N		Protease	675663052	XP_008996656	109 kDa	1.67	0.61	0.00	0.36	0.11			3.88	1.13	0.00	1.82	0.00	0.00	0.00	0.00	
		23	Immunoglobulin heavy chain			20502592	AAM22540**	11 kDa	1.03	1.01	0.00	0.98	0.21			3.10	0.00	0.00	3.03	0.00	0.00	0.00	0.00	
		24	Complement component C9			675646263	XP_002745089	65 kDa	1.03	0.81	0.00	0.78	0.21			3.10	0.00	0.00	2.43	0.00	0.00	0.00	0.00	

Only proteins that showed a significant change during pregnancy are shown; all protein data is shown in Supplemental Table 3.

Category 1: Proteins whose relative amount more than doubled or was undetectable initially but increased to ≥ 1 from the base level during pregnancy. Category 1a: Graph peak at second trimester. Category 1b: Graph peak at third trimester. Category 2: Proteins whose relative amount decreased to less than half or was initially detectable but decreased to undetectable levels during pregnancy. Category 2a: Graph minimum at second trimester. Category 2b: Graph minimum at third trimester. *: UniProtKB No., **: GenBank No.

Regarding molecules other than PRPs, several proteases and immunoglobulins (Igs) decreased, except for the Ig heavy chain variable region, while protein inhibitors and apolipoproteins increased, but there were no molecules in common between marmoset and human. Complements were also changed, but there were no common kinetics among them.

Collectively, an increase of protease inhibitors and a decrease of proteases were observed in both humans and marmosets during pregnancy. A2ML1 is predicted to be a PRP in marmoset and may have a function similar to that of PZP in humans.

### Kinetics of A2M family proteins in pregnancy

**Figure 2:**
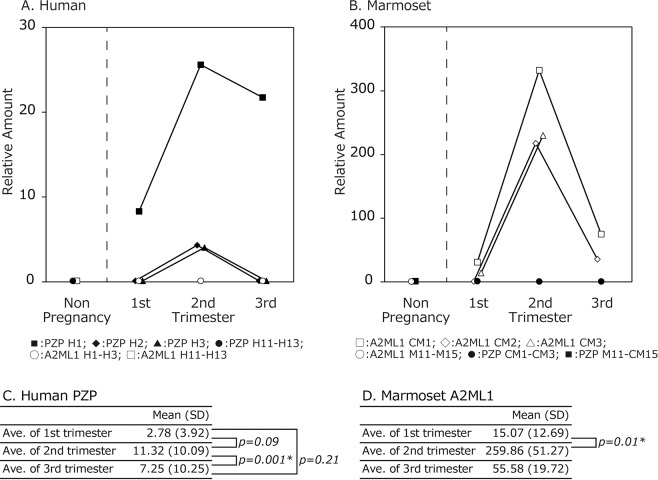
Kinetics of the relative amount of PZP and A2ML1 proteins in the sera of pregnant females. (**A**) PZP and A2ML1 relative amount detected by LC/MS in human serum. Relative amounts were compared in each trimester. Closed square: PZP (H1); closed diamond: PZP (H2); closed triangle: PZP (H3); closed circle: PZP (H11-H13); open circle: A2ML1 (H1-H3); closed square: A2ML1 (H11-H13). Sera were collected serially in each trimester of H1 to H3. (**B**) PZP and A2ML1 relative amount detected by LC/MS in marmoset serum. Relative amounts were compared in each trimester. Open square: A2ML1 (M1); open diamond: A2ML1 (M2); open triangle: (M3); open circle: A2ML1 (M11-M15); closed circle: PZP (M1-M3); closed square: PZP (M11-M15). Sera were collected serially in each trimester of M1 to M3. M3 had a premature birth and the data were not obtained. (**C**) Statistical analysis in human and (**D**) marmoset showing means and standard deviations of human PZP and marmoset A2ML1. *represents statistical significance.

As the expression of PZP and A2ML1, both of which are part of the A2M protein family, was complementary between human and marmoset pregnancies, the relative plasma concentrations of these proteins were plotted for each individual to examine the kinetics of A2M proteins in the plasma during pregnancy (Fig. 2).

PZP was detected from the first to third trimester in humans, while A2ML1 was not detected. Conversely, A2ML1 was detected during marmoset pregnancy, while PZP was not detected. The concentrations of both human PZP and marmoset A2ML1 increased at the second pregnancy trimester in all females, although concentrations varied significantly among samples. For example, PZP was undetectable in the two first-trimester samples and increased in the second trimester (relative amount = 4.0) in human plasma, and one sample had a higher score than the other two (8.32 and 25.59 in the first and second trimester, respectively). In marmoset, the concentration of A2ML1 showed a greater relative increase during pregnancy than that of PZP in human pregnancy. A2ML1 was undetectable in a first-trimester sample and increased greatly to 217.41 in the second trimester. The other two samples increased from 31.05 and 14.15 in the first trimester to 331.99 and 230.18, respectively, in the second trimester. Neither PZP nor A2ML1 was detected in non-pregnant humans or marmosets, respectively. The concentration of A2M was maintained at high levels without further increase in the second trimester of both human and marmoset pregnancies (Supplemental Table 1).

These results suggested that human PZP and marmoset A2ML1 have unique but common kinetics in the plasma during pregnancy, although the increase varied among individuals.

### Expression and localisation of PZP and A2ML1 in the placenta

Previous reports have described PZP expression throughout the body that flowed into the placenta and accumulated in the human intervillous space^17,30,31^. However, it was not clear whether A2ML1 also localises in the placenta of marmoset primates. Histological analysis was performed to investigate this phenomenon (Fig. 1E–H). Approximately half of the villous mesenchymal cells, almost all of syncytiotrophoblasts, and some decidual cells were stained with anti-human PZP antibody in human placenta specimens (Fig. 1F). Moreover, fibrous or agglomerate PZP was detected in the intervillous space and villous mesenchymal capillary, and A2ML1 was detected in the amnion of human placenta specimens (Fig. 1H). Contrastingly, marmoset PZP and A2ML1 could not be detected by IHC because of high cross-reactivity with other tissue regions (data not shown). Therefore, localisation of these proteins in the placenta could not be confirmed in marmoset.

To examine whether these molecules were expressed in human or marmoset placenta, mRNA expression was analysed by semi-quantitative RT-PCR. The placental chorion, decidua, and amnion tissues without disease at the third trimester were dissected and analysed. The relative expression levels of PZP and A2ML1 in these samples (Fig. 1I,J, Supplemental Fig. 2) were divided into four stages (Supplemental Table 2). PZP and A2ML1 mRNAs were expressed in all human specimens at varying degrees. However, the level of PZP mRNA tended to be lower than that expected based on IHC results, suggesting that the origin of intravillous PZP was not the placenta. Expression of A2ML1 in marmoset placenta also varied among specimens and sections of the placenta. Expression of PZP was not observed in marmoset. Expression of A2ML1 mRNA in the human placenta tended to be higher in the decidua than that in the chorion, whereas in the marmoset placenta, it tended to be higher in the chorion than that in the decidua. Collectively, although there were differences between humans and marmosets, PZP and A2ML1 mRNA were expressed in the third trimester placenta and were observed not only in the sera, but also in the chorion and decidua.

### Homology of PZP and A2ML1

**Figure 3:**
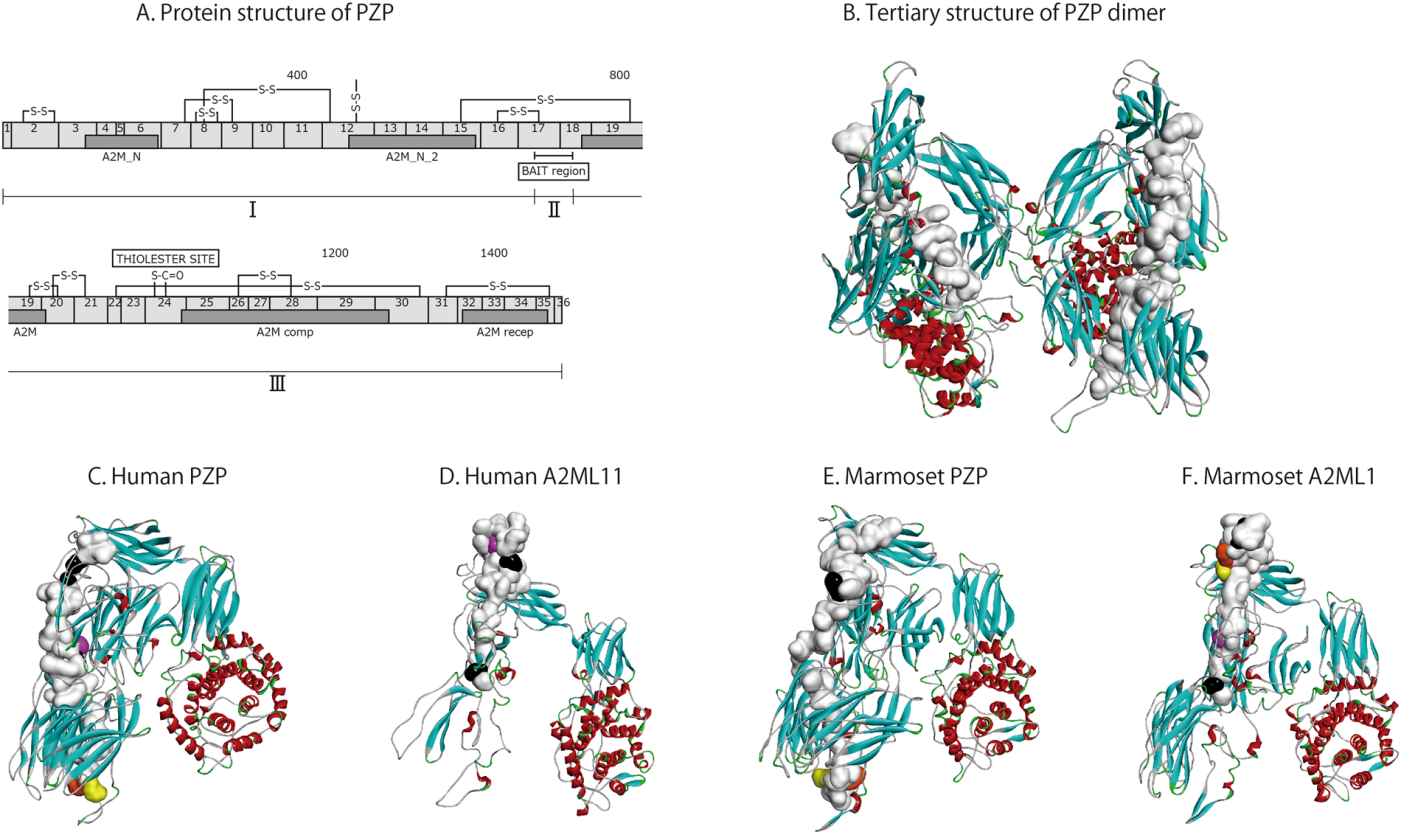
Tertiary structures of PZP and A2ML1. The structure of human PZP was predicted based on published amino acid sequences (NP_002855.2 and P20742) (**A**). A2M_N: Macroglobulin domain of alpha-2-macroglobulin; A2M_N_2: Alpha-2-macroglobulin family N-terminal region; A2M: Alpha-2-macroglobulin family C-terminal region; A2M_comp: A-macroglobulin complement component; A2M_recep: A-macroglobulin receptor; BAIT region: protease binding regions; Thiol-ester site: sequence that shows conformational change after protease binding; S-S: disulfide moiety; boxed numerals (1–36): exon number; 400-800-1200-1400: amino acid location. I: Amino acid sequence upstream of bait region; II: amino acid sequence of bait region; III: amino acid sequence downstream of C-terminal region after the bait region. Panels B–F: Predicted tertiary structures of human PZP dimer (**B**), human PZP monomer (**C**), human A2ML1 monomer (**D**), marmoset PZP monomer (**E**), and marmoset A2ML1 monomer (**F**). Different areas are represented by the following colours: Bait region, white; Proline-endopeptidase cutting site, pink; Aspartic acid-N endopeptidase cutting site, black; LysC, orange; LysN, yellow.

Although A2M proteins share common structural characteristics, the main function of these proteins, particularly that of A2ML1, has not yet been elucidated. Therefore, the common structure of the PZP and A2ML1 proteins was predicted based on the NCBI-enrolled sequence data (Supplemental Fig. 3). The representative human PZP scheme and sequence alignment are shown in Fig. 3A. The arrangement of each functional region including the macroglobulin domain is present in all PZP and A2ML1 proteins in both species. A unique bait region and thiol-ester site are also present in all human and marmoset PZP and A2ML1 proteins. These results demonstrated a high degree of conservation between the PZP and A2ML1 proteins in humans and marmosets (89.9 and 90.6% homology, respectively). Identity between PZP and A2ML1 proteins was about 70% in both humans and marmosets.

The bait region in PZP and A2ML1 is a unique region containing multiple protease cleavage sites that can be accessed by proteases that cleave the intermolecular thioester bond. Cleavage induces a conformational change that inactivates the proteases^32–34^. The sequence of the PZP and A2ML1 bait regions determines the accessibility and reactivity of the proteases. The bait region occupies about 3–4% of the total length of each protein, which is only a very small area (Fig. 3A; Table 2). BLASTp showed 60.7% and 56.8% identity between the bait regions of human and marmoset PZP and A2ML1, respectively, whereas BLASTn showed 77.4% and 74.2% identity between the bait regions of human and marmoset PZP and A2ML1, respectively. There was no identity between the bait regions of PZP and A2ML1 in either the humans or marmosets. The identity of other portions of the proteins is shown in Table 2. Notably, the bait regions had significantly lower homology between the overall PZP and A2ML1 sequences from humans and marmosets.

**Table 2:** Homology of PZP and A2ML1 bait region nucleotide (A) and amino acid (B) sequences between humans and marmosets.

A. Nucleotide				B. Protein			
PZP	(%)	homology (%)	*	PZP	(%)	homology (%)	**
Total (I–III)	100	92.5	4268/4612	Total (I–III)	100	89.9	1340/1491
(I)	45.1	91.4	1899/2078	(I)	45.7	89.9	613/682
(II)	3.6	77.4	130/168	(II)	3.8	60.7	34/56
(III)	51.3	94.6	2239/2366	(III)	50.5	92.0	693/753
**A2ML1**	**(%)**	**homology (%)**	*	**A2Ml1**	**(%)**	**homology (%)**	**
Total (I–III)	100	92.3	4650/5040	Total (I–III)	100	90.6	1187/1310
(I)	39.7	93.9	1879/2002	(I)	41.4	911.8	488/542
(II)	2.6	74.2	98/132	(II)	3.3	56.8	25/44
(III)	57.7	92.0	2673/2906	(III)	55.3	93.1	674/724

Each region (total: I–III, I, II, and III shown in Fig. 3A) was compared between species.

*matching marmoset and human nucleotides; **matching marmoset and human amino acids.

Despite a high degree of conservation between the protein sequences of PZP and A2ML1 in humans and marmosets, the homology of the bait region in each protein was lower depending on the species of origin and protein, and was not conserved in humans and marmosets.

**Figure 4:**
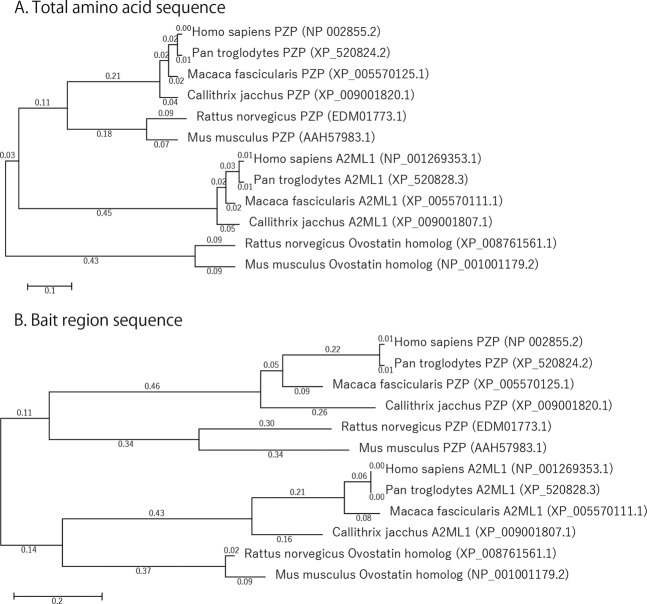
Phylogeny of PZP and A2ML1 proteins from primates and rodents inferred using MEGA6.06. Total amino acid sequences (**A**) and bait region sequences (**B**) were used for the comparison. NCBI sequence accession numbers are indicated. Numbers at branches indicate bootstrap values. Branch lengths represent the amount of change estimated to have occurred between nodes.

To understand the evolution of the PZP and A2ML1 bait regions in primates, phylogenetic trees were constructed using the amino acid sequences of the whole proteins and bait regions (Fig. 4). Both trees were divided into two main lineages, PZP and A2ML1, but the phylogenetic distance between the whole sequences was shorter than that between the bait regions alone. In particular, the genetic distances between human and marmoset in the bait region were 3–6 times greater than those of the whole region (0.04 and 0.05 over the sequence and 0.26 and 0.16 over the bait region in PZP and A2ML1, respectively). These data confirmed the unique evolution of the bait region, indicating that the specificity of these proteins as protease inhibitors might differ between humans and marmosets.

Analysis of single nucleotide polymorphisms (SNPs) in human PZP and A2ML1 using the NCBI SNP GeneView tool revealed 52 SNPs for which the minor allele frequency (MAF) is ≥1% in the complete mRNA sequence of human PZP, of which 29 were missense mutations (Supplemental Fig. 4A). The bait region alone showed 4 SNPs, one of which was a missense mutation. SNPs resulting in amino acid substitutions were present at a rate of 0.76% and 1.75% in the PZP sequence and the bait region, respectively, implying that amino acid substitutions were more frequent in the bait region.

Similar to the human PZP, 61 SNPs for which the minor allele frequency (MAF) is ≥1% have been reported in the complete mRNA sequence of human A2ML1, 45 of which are missense mutations, including one nonsense and two frameshift mutations (Supplemental Fig. 4B). In A2ML1, SNPs with amino acid substitutions were present at a rate of 1.26% and 1.67% in the whole A2ML1 mRNA sequence and the bait region, respectively. As with PZP, amino acid substitution seems to occur more frequently in the A2ML1 bait region. SNPs and protease cutting sites (CSs) in the bait region of human PZP and A2ML1 are shown in Supplemental Fig. 4C,D. Those protease CSs were not affected.

SNP analysis suggested that amino acid substitutions are more likely to accumulate in the bait region of PZP and A2ML1 than in other protein regions.

### Prediction of PZP and A2ML1-associated molecules

The diversity of amino acid sequences in the bait regions of PZP and A2ML1 suggested that the accessibility and cleavage sites of proteases might be altered, but further analyses found that few protease CSs were changed (Table 3). In total, nine proteases were predicted to react with CSs common to PZP and A2ML1 in humans and marmosets and four proteases were expected to have different reactions in humans and marmosets. The changed site contained proline-endopeptidase (PE), aspartic acid-N endopeptidase (Asp-N E), lysyl endopeptidase (LysC), and peptidyl-Lys metalloendopeptidase (LysN) detected by peptide cutter application (https://web.expasy.org/peptide_cutter/). Lys proteases are trypsin-like proteases and aspartic proteases contain retrovirus and retrotransposon digestive proteases, which may be important for pathogen digestion *in vivo*.

**Table 3:** Protease accessibility to bait region of each protein.

	Human		Marmoset	
	PZP	A2ML1	PZP	A2ML1
Aspartic acid-N endopeptidase	−	+	+	+
Aspartic acid-N endopeptidase + N-terminal glutamic acid	+	+	+	+
Chymotrypsin	+	+	+	+
Clostripain	+	+	+	+
Glutamyl endopeptidase	+	+	+	+
Lysyl endopeptidase	+	−	+	+
Peptidyl-Lys metalloendopeptidase	+	−	+	+
Pepsin	+	+	+	+
Prolyl-endopeptidase	+	+	−	+
Proteinase K	+	+	+	+
Staphylococcal peptidase I	+	+	+	+
Thermolysin	+	+	+	+
Trypsin	+	+	+	+

PeptideCutter was used for *in silico* prediction of protease cleavage sites in protein sequences. Predicted cutting sites of each protease are shown in Supplemental Fig. 4.

To confirm the position of the bait region and CS, three-dimensional structures of human and marmoset PZP and A2ML1 were constructed by homology modelling based on the crystal structure of human A2M (PDP ID:4ACQ). The structure of human PZP predicted by homology modelling is similar to previously reported structures (Fig. 3B). It is speculated that one bait region of the human PZP dimer is exposed to the outside of the protein but the other is located inside of the protein, making only one PZP-bait region exposed to proteases. Contrastingly, CSs of PE, Asp-N E, LysC, and LysN were exposed on the surface of each protein. (Fig. 3C–F).

## Discussion

This study aimed to clarify which molecules are expressed and circulated in primate pregnancy and to describe the functional importance of these molecules by comparative analysis of PRP expression in humans and marmosets. The human, marmoset, and murine immune systems are evolutionarily equidistant. However, evolution of the placenta has been rapid, and highly invasive trophoblasts have evolved comparatively recently in the primate lineage. As trophoblast invasion in new-world monkeys had not been clearly identified, we first confirmed that the marmoset has invasive villi in the placenta using a transgenic GFP marmoset foetus and a WT mother’s placenta (Fig. 1). Because marmosets construct a maternal-foetal interface that is maintained for a longer period than that of rodents, marmosets might present an advantage compared to mice as a model animal to investigate the molecular mechanism of maternal immune tolerance against foetal tissues in different trimesters.

It is known that marmoset, which often conceives multiple twins, has anastomosis in the placenta, resulting in haematopoietic chimerism^35^. Contrastingly, in the placenta of human twins, the blood is scarcely mixed directly. Therefore, it is plausible that a finer-tuned function of immune suppression is required in marmoset compared to humans (Fig. 2), and marmoset A2ML1 increased to a higher plasma concentration than that of human PZP during pregnancy. This evidence suggests that high A2ML1 level in marmoset is required for the suppression of mother’s immunity during pregnancy.

Regarding the immune suppression mechanism, LC-MS analysis revealed that different PRPs, PZP and A2ML1, were increased depending on the species. However, for proteins other than PRPs, there were no common ones showing the same kinetics, and only several proteins sharing similar characteristics such as proteases and protease inhibitors showed similar kinetics (Table 1). Human proteases decreasing in the second trimester were plasma kallikrein and carboxypeptidase N. Contrastingly, marmoset proteases decreasing in the second trimester were aminopeptidase N and prasminogen. These proteases are serine proteases and aminopeptidases. The protease recognition sites of PZP and A2ML1 bait regions contain such protease inhibitory sites, which indicated that PZP and A2ML1 might influence the decrease of these proteases in human and marmoset sera, respectively. However, as proteases and protease inhibitors physiologically antagonise each other, PZP and A2ML1 might regulate different proteases since they share few common proteases and protease inhibitors. Moreover, reduced proteins involve proteases that destroy and reorganise their tissues, which make the environment protective because of a decrease in proteases and an increase in several protease inhibitors. Therefore, even if foetal fragments flow into the maternal blood during pregnancy, inflammation may be suppressed in the maternal tissue.

On the other hand, in our study, other human protease inhibitors increased in pregnancy included several serpins such as anti-thrombin-III (SERPINC1), which suppresses coagulation of blood, and alpha-1-antichymotrypsin (SERPINA3), which suppresses cathepsin G in neutrophils and chymases in mast cells to protect tissues from inflammation. SERPINA3 was reported to reach the maximal serum concentration at 26–30 weeks of gestation^36^, and SERPINC1 was reported to show no difference in plasma concentrations between 12–14 weeks and 24–28 weeks of gestation^37^, but the individual differences were large in our results. In marmoset, plasma serine protease inhibitor levels increased in pregnancy. Compared to that in marmoset, human protease inhibitor targets innate immune cells more specifically, collaborating with lymphocytes of acquired immunity. This might suggest a difference in human and marmoset acquired immunity. As we reported previously^25^, marmoset has a lower B cell response and less Igs, which may indicate that the risk of IgE and mast cell-dependent inflammation or neutrophil reactions with opsonisation of IgGs is lower in this species. These results suggested that several serum proteins other than PZP and A2ML1 may substitute their function.

Serine protease inhibitor (SERPIN) family proteins include not only protease inhibitors but also regulators of coagulation or fibrinolysis, transporters of hormones, and suppressors of inflammation^38^. It is known that abnormalities of SERPIN are associated with preeclampsia, a condition in which abnormal blood vessels form in the placenta and become ischaemic. Vascular endothelial cells are damaged in this condition, causing hypertension and high proteinuria. An increase of the PAI-1 (SERPINE1)/PAI-2 (SERPINB2) ratio was reported to be one of the predictors^39^. Some functions of serpins other than protease inhibition may be involved in the cause of the disease. PZP, which we focused on, has a similar function to serpins and showed more drastic changes during pregnancy. Thus, functions of PZP or A2M family proteins other than protease inhibition might also be related to pregnancy-associated diseases such as preeclampsia. However, compared to that of SERPIN, PZP function is less understood.

PZP is produced in the liver, placenta, and other tissues, and its blood concentration increases during pregnancy. PZP mRNA has also been reported to be expressed in placenta in a small amount, about one-thousandth of that in the liver, which is one of the secretory sources in human^30^. However, it is not known whether the PZP protein observed in the intervillous space was secreted in the placenta or flowed into the placenta via maternal blood after being secreted in the liver. We examined PZP protein expression level of the placenta and serum during pregnancy by using the fresh placenta immediately after the scheduled caesarean section and maternal plasma of each trimester. Plasma concentration of PZP reached its peak in the second trimester and decreased significantly in the third trimester. Contrastingly, since placental function was maintained in the scheduled caesarean section, the expression level of PZP is unlikely to be changed by degradation of mRNA induced by collapse of the placenta. Additionally, the expression of PZP was mostly positive in the intervillous space (Fig. 1F). It is suggested that PZP was secreted in the liver and flowed into the placenta by maternal blood during pregnancy.

Furthermore, interferon and steroid response elements exist in the promoter region of PZP, which suggests PZP expression is regulated by interferon or steroid hormones^30^. Women with high oestrogen levels caused by hormone replacement have been reported to have high PZP levels^40^, suggesting that elevated oestrogen levels during pregnancy may regulate PZP levels.

Compared to A2M, PZP has a lower inhibitory activity toward some proteases. For example, while PZP is highly reactive to cellular protease, it is less reactive to trypsin. Contrastingly, A2M is highly reactive to both of these proteases^41^. Based on this, it was speculated that the affinity of protease inhibitors to target proteases can differ, even within a family of proteins.

Results from the current study, showing an increase of PZP during pregnancy, are consistent with previous reports^17,30^. Following our observation that both the human PZP and the marmoset A2ML1 markedly increased in plasma during the second trimester, we analysed the bait region of both molecules in detail. Moreover, we used predictive tertiary structure analyses to examine whether proteases could access the bait region from the outside of the protein (Fig. 3). The two trypsin CSs predicted by PeptideCutter were present at both ends of the bait region, which were difficult to access from the outside of both PZP and A2ML1, supporting previous reports^41,42^. Although the homology was not high between the human and marmoset bait regions of PZP and A2ML1, they had many target proteases in common. The proteases that were not shared included PE, Asp-N E, LysC, and LysN. PE is thought to be involved in the enhancement of inflammation by inducing collagen degradation and neutrophil migration^43^. Asp-N E, LysC, and LysN might affect bacterial infection and other degradation functions. Therefore, it might be possible that PZP and A2ML1 play a different role in some regulatory mechanism for inflammation and bacterial infection between human and marmoset species.

Phylogenetic analysis of known PZP and A2ML1 sequences showed that the total protein sequences were more highly conserved than those of the bait regions (Fig. 4 and Table 2). Comparing the phylogenetic tree of PZP/A2ML1 total sequences with that of haemoglobin α and β, which are two commonly referenced molecules in evolution (Supplemental Fig. 5), both proteins show the same degree of branching from humans to new-world monkeys. Contrastingly, the phylogenetic tree of the bait region, a small part of the whole PZP/A2ML1 molecule, has a longer branching distance than that of the total sequence and haemoglobins. The bait regions had high homology between humans and old-world monkeys, but a significantly lower homology with marmosets. The characteristics of marmoset pregnancy and placental formation likely affected evolution. Since marmosets are usually pregnant with twins with allogenic tissue-fused placenta, they might possess a unique immunosuppressive mechanism that is different from that of humans and old-world monkeys, which have a single placenta. Further elucidation of the sequences of the PZP and A2ML1 bait regions of other primates could help to address this issue in the future.

In our results, mutations and SNPs were accumulated within the bait region, resulting in the low homology observed. Nevertheless, no significant difference was observed in the protease CSs, and reactivity was not changed even where amino acid substitution had occurred within CSs. Although the SNP accumulation mechanism is not clear, protease inhibition is essential for the maintenance of pregnancy and is also related to speciation, so it might be conserved in the process of primate evolution, as significant changes to protease regulation that would threaten species maintenance would be counteracted by evolution.

Our SNP analysis was limited to MAF ≥ 1%. However, those with MAF < 1% or some unreported SNPs may also affect protease inhibitory function, as such mutations may create or destroy CSs. As a result, an increase of autoimmune disease may be induced. Therefore, the accumulation of mutations might be evolutionally avoided.

Apart from pregnancy disorders, increase of serum PZP has also been reported in Alzheimer’s disease^44^, and an increase of A2ML1 was reported in paraneoplastic pemphigus^45^. In these diseases, local inflammation and accumulation of immune cells were reported. Evidence from these studies indicated that PZP and A2ML1 play an important role not only in pregnancy, but also in other inflammatory diseases. The involvement of the PZP and A2ML1 bait regions in inflammatory diseases related to pregnancy and other incurable diseases warrants further investigation.

Finally, we confirmed that marmoset is a useful experimental animal to explore human gestational immunity, as marmoset has invasive trophoblasts and prolonged gestation. Plasma concentrations of human PZP and marmoset A2ML1 were increased during pregnancy. These molecules have multiple CSs of various proteases exposed to the molecular surface, including CSs for a common proinflammatory protease, PE. Systemic and local regulation of the proteinases by PZP and/or A2ML1 molecules may be important in gestational immunity.

## Methods

### Clinical samples and animal tissues

All samples in this study are listed in Supplemental Table 3. In accordance with the informed consent and research protocol approved by Tokai University IRB (09R-056), human maternal plasma was collected at the first stage (12 to 14 weeks), second stage (20 to 22 weeks), and third stage (35 to 37 weeks) of pregnancy. A part of the human placenta was collected after delivery by planned caesarean section. Marmoset samples were provided by the Central Institute for Experimental Animals (CIEA). According to the research protocol approved by CIEA IRB (14029 A, 15020 A, 16019 A), marmoset maternal plasma was collected at the first stage (50 days), second stage (100 days), and third stage (120 days) of pregnancy. These stages are considered comparable to the first, second, and third trimesters. Marmoset placenta was collected after delivery by planned caesarean section. GFP-WT chimeric placenta was obtained from WT mother marmosets delivering GFP marmoset new-borns. Marmoset tissue sections for IHC or RNA extraction were cut out and treated within 2 h of delivery. All methods were carried out in accordance with relevant guidelines and regulations for both human and animal studies.

### Liquid chromatography-ion trap mass spectrometry (LC-MS/MS) analysis

LC-MS/MS was performed according to the protocol we reported previously^46,47^. Plasma (2 µl) was diluted with 100 µl of 50 mM TEAB and mixed with 10 µl of 200 ng µL^−1^ trypsin solution. After digestion at 37 °C for 20 h, 5 µl of 100 mM dithiothreitol (DTT) was added to the digests. This mixture was incubated at 95 °C for 5 min and dried using a Speed Vac (Thermo Scientific, Inc., Waltham, MA, USA). For LC-MS/MS analysis, digests were dissolved into 50 µl of 0.1% trifluoroacetic acid (TFA) and applied to an UltiMate 3000 HPLC system (Thermo Scientific, Inc.). A reverse-phase column (Develosil 300ODS-HG-5; 1.0 mm i.d. × 100 mm; Nomura Chemical Co. Ltd., Seto, Japan) was used at a flow rate of 50 µl min^−1^ with a 0–60% linear gradient of acetonitrile in 0.1% formic acid. Eluted tryptic peptides were subjected directly to QExactive hybrid quadrupole-Orbitrap mass spectrometry (Thermo Scientific Inc.), with spray voltage set at 1.9 kV and collision energy at 30%. The mass acquisition method consisted of one full MS survey scan followed by an MS/MS scan of the most abundant precursor ions from the survey scan. Dynamic exclusion of MS/MS spectra was set to 30 s. An MS scan range of 300–2000 m/z was employed in the positive ion mode, followed by data-dependent MS/MS of the top five ions in order of abundance. Data were analysed with Proteome Discoverer (Thermo Scientific Inc.), Mascot (Matrix Science Inc., Boston, MA, USA), and Scaffold (Proteome Software Inc., Portland, OR, USA). The NCBI database (GenBank) was used (https://www.ncbi.nlm.nih.gov/), and tandem mass spectra (MS/MS) were searched against the SwissProt database (https://www.uniprot.org/) with the following parameters: monoisotopic mass, peptide mass tolerance of 10 ppm, fragment ion mass tolerance of 0.8 Da, complete tryptic digestion allowing two missed cleavages, variable modifications of methionine oxidation. Scaffold was used for label-free quantitation. Peptide and protein probability thresholds of 90% and 99%, respectively, were applied. Using a target decoy approach, a peptide false discovery rate (FDR) of 0.2% was determined. The relative amount was calculated by LC-MS based on albumin (Alb), which did not change prominently during pregnancy.

### Immunohistochemistry

Sections of formalin-fixed paraffin-embedded tissues were stained using two polyclonal antibodies for GFP (GFP antibody, clone #NB600–308SS; Novus Biologicals. Littleton, CO, USA), human PZP (anti-human PZP antibody, clone #HPA041471; Atlas Antibodies, Bromma, Sweden), and human A2ML1 (anti-human A2ML1 antibody, clone #HPA038848; Atlas Antibodies) as the primary antibody. Anti-rabbit IgG-HRP (CSA II Rabbit Link, #K1501; Agilent Technologies, Inc., Santa Clara, CA, USA) was used as the secondary antibody.

For the reaction enhancement, Amplification Reagent (CSA II Biotin-free Tyramide Signal Amplification System, #K1497; Agilent Technologies, Inc.) was used. Sections were stained using diaminobenzidine (Wako Chemicals Dotite DAB, #347–00904; Dojindo Laboratories. Kumamoto, Japan) solution according to the manufacturer’s instructions. Rabbit IgG (Rabbit Immunoglobulin Fraction, #X0936; Agilent Technologies, Inc.) was used instead of the primary antibodies for negative controls.

### RT-PCR and DNA sequence analysis

The RNA extraction and RT-PCR were performed based on the conditions we reported previously^48^. Total RNA was extracted using TRIzol LS reagent (Invitrogen, San Diego, CA, USA) from fresh-frozen tissues according to the manufacturer’s instructions. The amount of extracted RNA used for RT-PCR was calculated based on the concentration as determined by optical density. The primers of human PZP, human A2ML1, marmoset PZP, marmoset A2ML1, and β-actin are shown in Supplemental Table 4. The conditions of cDNA amplification involved initial reverse transcription at 50 °C for 30 min and 95 °C for 15 min, followed by 35 cycles of denaturation for 1 min at 94 °C, annealing for 1 min at 60 °C, and extension for 1 min at 72 °C, and final extension for 5 min at 72 °C. After the reaction, PCR products were separated by electrophoresis on a 2% agarose gel in Tris-borate-EDTA (TBE) buffer and stained with 0.5 µg mL^−1^ ethidium bromide.

Direct DNA sequencing of the PCR products was performed using the same primers and an Applied Biosystems 3500XL sequencer (Applied Biosystems, Carlsbad, CA, USA). Each sequence was compared with those registered in the NCBI database, using A Plasmid Editor (ApE, by M. Wayne Davis).

### Tertiary structure analysis

Three-dimensional structures of PZP and A2ML1 were constructed by homology modelling based on the crystal structure of A2M deposited at the Protein Data Bank (https://www.rcsb.org/). The four distinct, independent molecules in the crystal structure of A2M (PDB ID: 4ACQ) take essentially identical structures with the root-mean square deviation of the Cα atoms being 0.854 Å. Therefore, the structure of the A chain was used as the template. Homology modelling of PZP and A2ML1 was performed using the segment matching algorithm^49^ implemented in a Molecular Operating Environment (MOE) (http://www.chemcomp.com). Multiple intermediate structures were constructed. The structures with the best GBVI/WSA_dG^50^ scoring function were selected and optimised.

### Prediction of protease cleavage sites and sequence analyses

PeptideCutter (Swiss Institute of Bioinformatics; https://web.expasy.org/peptide_cutter/) was used for comprehensive *in silico* prediction of protease CS in the bait region of each protein. ClustalW2 (EMBL-EBI Hinxton, UK; https://www.ebi.ac.uk/Tools/msa/clustalw2/) was used for protein structure alignment. MEGA6.06 (MEGA; https://www.megasoftware.net/) was used for phylogenetic inference.

### Statistical analyses

Microsoft Excel (Microsoft Office 2016, Microsoft Corporation, Redmond, WA) was used for data management and t-test analysis. SIMCA 13.0 (Sartorius Stedim Biotech, Goettingen, Germany) was used for PCA.

## Supplementary information

Supplementary information.

## Data Availability

All data generated or analysed during this study are included in this published article and its Supplementary Information Files.
