# Supplementary material for: Human PZP and common marmoset A2ML1 as pregnancy related proteins
Source: Sci Rep. 2020 Mar 20;10:5088. doi: 10.1038/s41598-020-61714-8 (PMC7083932; doi:10.1038/s41598-020-61714-8)
Supplement: Supplementary file 1 — Supplementary information. [file 41598_2020_61714_MOESM1_ESM.pdf]

Supplementary Information

Human PZP and common marmoset A2ML1

as pregnancy related proteins

Hirofumi Kashiwagi, Hitoshi Ishimoto, Sunichiro Izumi,
Toshiro Seki, Rihito Kinami, Asako Otomo,
Kazumi Takahashi, Fuyuki Kametani, Noriaki Hirayama,
Erika Sasaki, Takashi Shiina, Kou Sakabe,
Mikio Mikami, Yoshie Kametani*

Supplemental Fig. 1

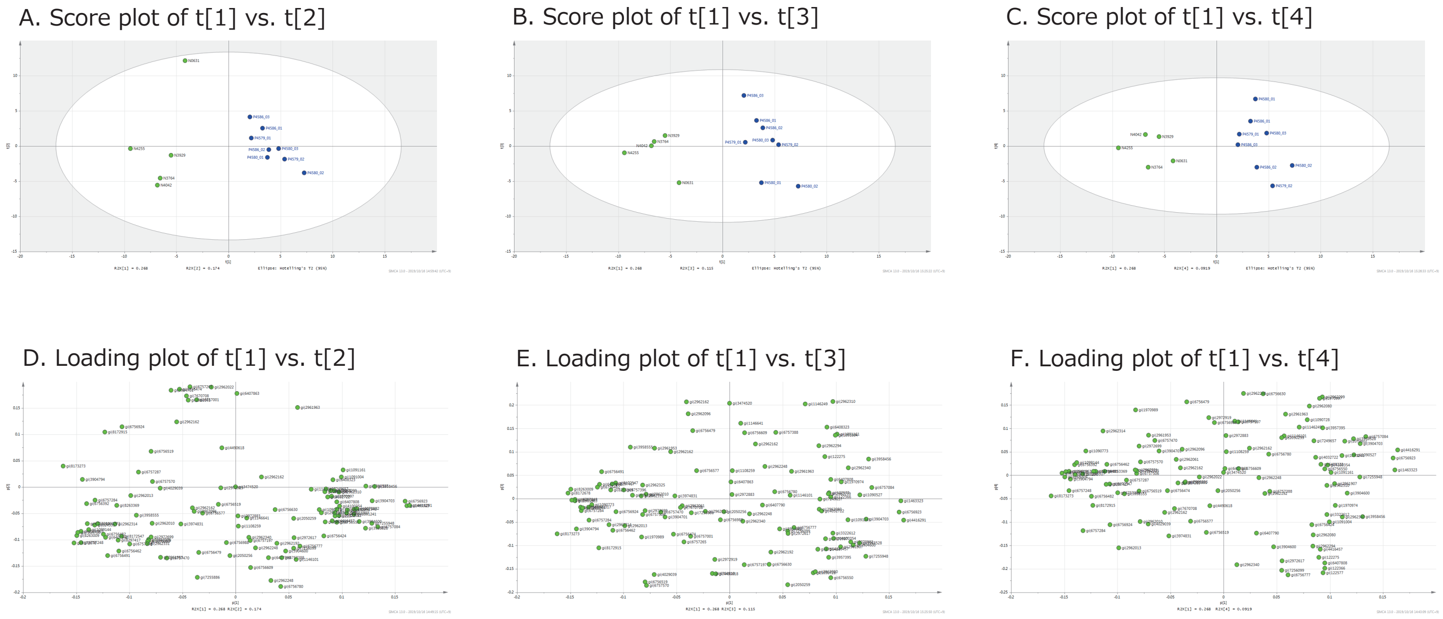

Supplemental Fig. 1. Principal Component Analysis. t-Predicted score scatterplot showing the results for samples prepared from pregnant and non-pregnant marmoset sera. Blue: pregnant samples; Green: non-pregnant samples. R2X[1] = 0.268; R2X[2] = 0.174; R2X[3] = 0.115; R2X[4] = 0.0919; Ellipse: Hotelling' s T2 (95%).

Each sample name conforms to Supplemental Table 3. Principal component analysis was performed using SIMCA 13.0 (Sartorius Stedim Biotech Goettingen, Germany).

Supplemental Fig. 2

A.

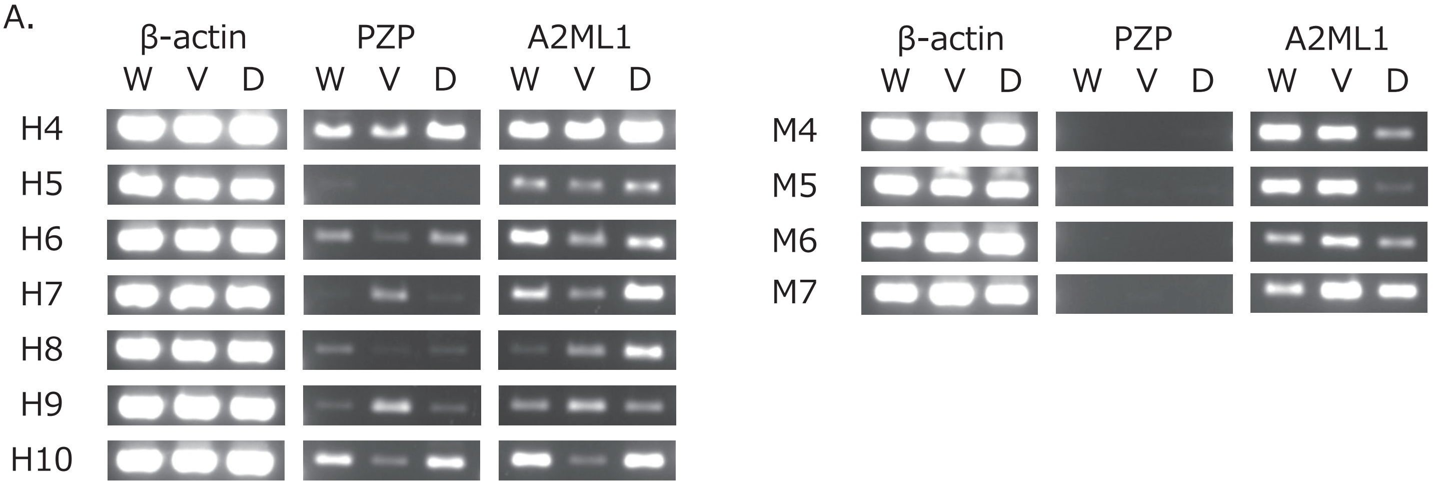

B.

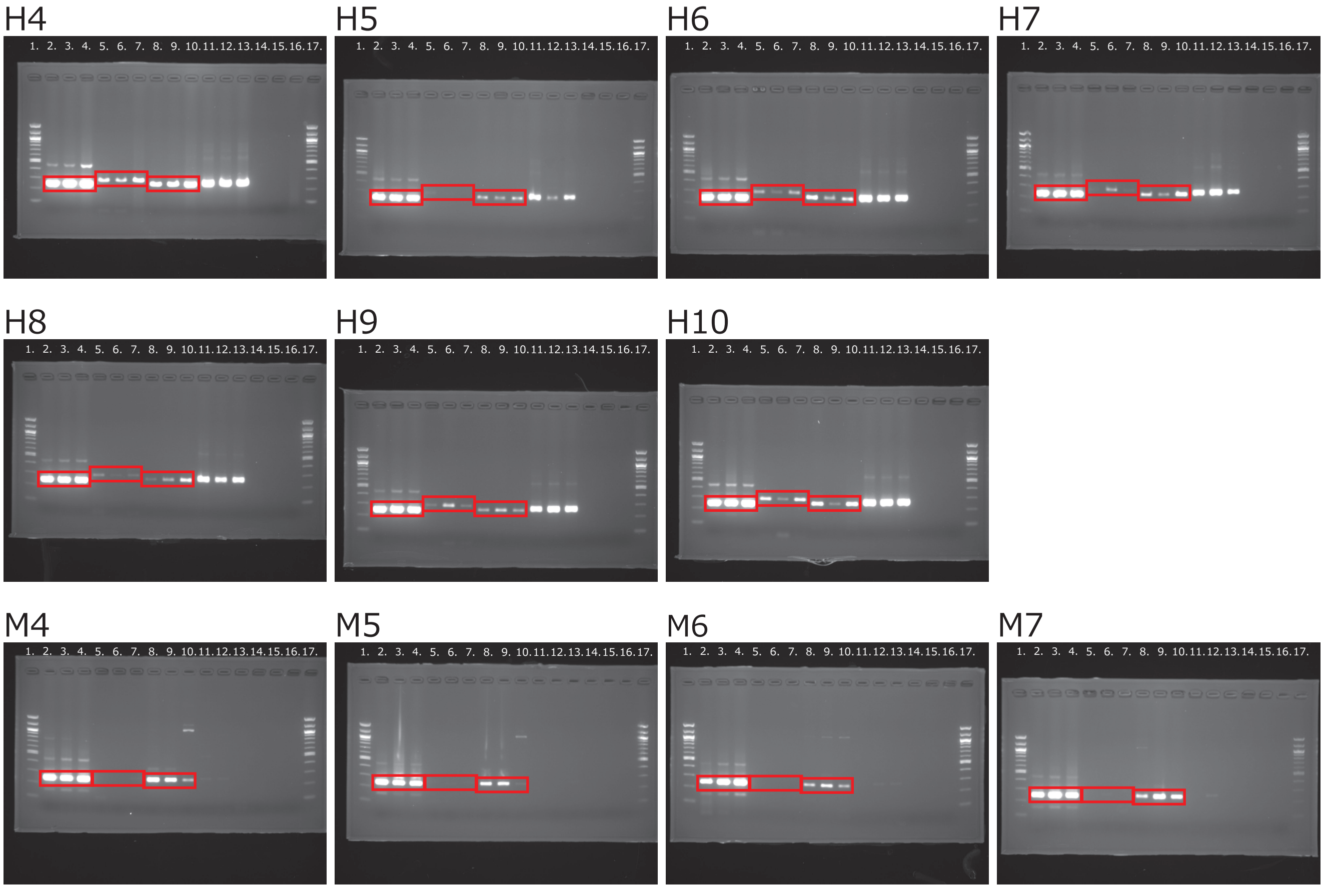

Supplemental Fig. 2. Tissue-specific expression of PZP and A2ML1 mRNA detected by semi-quantitative RT-PCR.
A. Comparison of the band intensity of PZP and A2ML1
W: whole placenta; V: villi; D: decidua; β-actin: positive control
B. Full images of gel electrophoresis
Lane1: 100bp DNA Ladder Maker; Lane2: whole Placenta β-actin; Lane3: villi β-actin; Lane4: decidua β-actin; Lane5: whole Placenta PZP; Lane6: villi PZP; Lane7: decidua PZP; Lane8: whole Placenta A2ML1; Lane9: villi A2ML1; Lane10: decidua A2ML1; Lane11: whole Placenta PSG; Lane12: villi PSG; Lane13: decidua PSG; Lane14: whole Placenta Negative control; Lane15: villi Negative control; Lane16: decidua Negative control; Lane17: 100bp DNA Ladder Maker.
PSG: pregnancy specific beta-1-glycoprotein 5, data not used in this study.

Supplemental Fig.3

```
PZP_h_NP_002855.2            -MRKDRLLHLCLVLLLILLSASDSNSTEPQYMVLVPSLLHTEAPKKGCVL 49
PZP_CM_XP_009001820.1        -MGKDRRLHLCLMLLLLLLSANDSDSAEPQYMVLVPSLLHTETPEKGCVL 49
A2ML1_h_NP_001269353.1       --------------------------------------------------
A2ML1_CM_XP_009001807.1      --------------------------------------------------
A2M_h_NP_000005.2            MGKNKLLHPSLVLLLLVLLPTDASVSGKPQYMVLVPSLLHTETTEKGCVL 50
A2M_CM_XP_009001819.1        -MGKNKLLHLSLVLLLVLLPTEASDSGKPQYMVLVPSLLHTETAEKCCIL 49

PZP_h_NP_002855.2            LSHLNETVTVSASLESGRENRSLFTDLVAEKDLFHCVSFTLPRISASSEV 99
PZP_CM_XP_009001820.1        LSHLNETVTLSASLESARGNRSLFTDLVAEKDLFQCVSFTLPRISASSEV 99
A2ML1_h_NP_001269353.1       --------------------------------------------------
A2ML1_CM_XP_009001807.1      --------------------------------------------------
A2M_h_NP_000005.2            LSYLNETVTVSASLESVRGNRSLFTDLEAENDVLHCVAFAVPKSSSNEEV 100
A2M_CM_XP_009001819.1        MSYLKETVTVSASLESIRGNSSLFTDLVAETDVLHCVAFSVPKISSSEEV 99

PZP_h_NP_002855.2            AFLSIQIKGPTQDFRKRNTVLVLNTQSLVFVQTDKPMYKPGQTVRFRVVS 149
PZP_CM_XP_009001820.1        AFLSIQIKGPTQDFRKRNTVLLKNAQSLVFVQTDKPIYKPGQTVRFRIVS 149
A2ML1_h_NP_001269353.1       --------------------------------------------------
A2ML1_CM_XP_009001807.1      --------------------------------------------------
A2M_h_NP_000005.2            MFLTVQVKGPTQEFKKRTTVMVKNEDSLVFVQTDKSIYKPGQTVKFRVVS 150
A2M_CM_XP_009001819.1        MFLTVQVKGPTQEFKKRTSVIVKNDESLVFVQTDKSIYKPGQTVKFRVVS 149

PZP_h_NP_002855.2            VDENFRPRNELIPLIYLENPRRNRIAQWQSLKLEAGINQLSFPLSSEPIQ 199
PZP_CM_XP_009001820.1        VDENFHPQNELIPLIYIENPRRNRVAQWQSLKLEGGLNQLSFPLSSEPIQ 199
A2ML1_h_NP_001269353.1       --------------------------------------------------
A2ML1_CM_XP_009001807.1      -------------MVELQDPNSNRIAQWLEVVPEQGIVDLSFQLAPEAML 37
A2M_h_NP_000005.2            MDENFHPLNELIPLVYIQDPKGNRIAQWQSFQLEGGLKQFSFPLSSEPFQ 200
A2M_CM_XP_009001819.1        LDETFHPLNELIPLVYIQDPKGNRIAQWQSLELEGGLKQLSFPLSSEPFQ 199

PZP_h_NP_002855.2            GSYRVVVQTESGGRIQHPFTVEEFVLPKFEVKVQVPKIISIMDEKVNITV 249
PZP_CM_XP_009001820.1        GSYKVVVHTESGGRIEHPFAVEEFVLSKFEVEIQVPKIISIMDEKVNITV 249
A2ML1_h_NP_001269353.1       --------------------------------------------------
A2ML1_CM_XP_009001807.1      GTYTVAVAEG---KTFGTFSVEEYVLPKFKVEVVEPKELSTVQESFLVKI 84
A2M_h_NP_000005.2            GSYKVVVQKKSGGRTEHPFTVEEFVLPKFEVQVTVPKIITILEEEMNVSV 250
A2M_CM_XP_009001819.1        GSYKVVVQKESGGRREHPFTVEEFVLPKFEVQVTVPKIITILEEEMNVSV 249

PZP_h_NP_002855.2            CGEYTYGKPVPGLATVSLCRKLSRVLNCDKQE-----VCEEFSQQLNSNG 294
PZP_CM_XP_009001820.1        CGKYTYGKPVPGLATVSLCRKYFPVSYCAPKE-----ICEEFSQQLNSNG 294
A2ML1_h_NP_001269353.1       --------------------------------------------------
A2ML1_CM_XP_009001807.1      CSRYTYGKPMLGTVQVSVCQKAHTYWYREVEREQLSDKCRNLSGQTDKTG 134
A2M_h_NP_000005.2            CGLYTYGKPVPGHVTVSICRKYSDASDCHGEDSQA--FCEKFSGQLNSHG 298
A2M_CM_XP_009001819.1        CGLYTYGKPVPGHVTVSVCRKYNDASDCFGEESQV--YCEKFSGQLNSHG 297

PZP_h_NP_002855.2            CITQQVHTKMLQITNTGFEMKLRVEARIREEGTDLEVTANRISEITNIVS 344
PZP_CM_XP_009001820.1        CITQQVQANLLQIKNTGFEMKLRVEARIREEGTDVVVTTNGISEITNVAS 344
A2ML1_h_NP_001269353.1       --------------------------------------------------
A2ML1_CM_XP_009001807.1      CFSAPVDMATFNLTGYAYSHEINIVATVVEEGTGVEANATQSVYISPEMG 184
A2M_h_NP_000005.2            CFYQQVKTKVFQLKRKEYEMKLHTEAQIQEEGTVVELTGRQSSEITRTIT 348
A2M_CM_XP_009001819.1        CFSRQLKTKVFQLKRKEYEMKLHTEAKIQEEGTEVELTGRETSEITRTVT 347

PZP_h_NP_002855.2            KLKFVKVDSHFRQGIPFFAQVLLVDGKGVPIPNKLFFISVNDAN--YYSN 392
PZP_CM_XP_009001820.1        KLIFVKVDSHFRRGIPFFGQVLLVDGKGVPIPNKLIFISVNEAN--YHFN 392
A2ML1_h_NP_001269353.1       --------------------------------------------------
A2ML1_CM_XP_009001807.1      SMTFEDTNDFYHPNFPFSGKISVRGHDDSLLKNHLVFLVIYGTNGVLKQT 234
A2M_h_NP_000005.2            KLSFVKVDSHFRQGIPFFGQVRLVDGKGVPIPNKVIFIRGNEAN--YYSN 396
A2M_CM_XP_009001819.1        KLSFVKVDSNFRQGIPFFGQVRLVDGKGVPMPNKVVLIRANEAN--HHSN 395

PZP_h_NP_002855.2            ATTNEQGLAQFSINTTSISVNKLFVRVFTVHPNLCFHYSWVAEDHQGAQH 442
PZP_CM_XP_009001820.1        TTTNEQGLVQFSIDTTNILANNLFVTVSSERPSFCFPYSWITEVHQHAQH 442
A2ML1_h_NP_001269353.1       --------------------------------------------------
A2ML1_CM_XP_009001807.1      LVTDNDGLASFTLDTSSWNGTDVSLEGRFQMEDLVYNPGQVPRYYQNAYL 284
A2M_h_NP_000005.2            ATTDEHGLVQFSINTTNVMGTSLTVRVNYKDRSPCYGYQWVSEEHEEAHH 446
A2M_CM_XP_009001819.1        ATTDEHGLVQFSINTTNVMGTSLSVRVRYKDRHPCYTYQWVLEENEEAQH 445

PZP_h_NP_002855.2            TANRVFSLSGSYIHLEPVAGTLPCGHTETITAHYTLNRQAMGELSELSFH 492
PZP_CM_XP_009001820.1        TASHVFSLSGSYVDLEPVAGTLPCGHTQTIRAHYMLKRQAVRELSEFSFH 492
A2ML1_h_NP_001269353.1       --------------------------------------------------
A2ML1_CM_XP_009001807.1      HLRPFYNTTRSFLGIHRLSGPLKCGGQPQEVLVDYYIDPADASPDQEISFS 334
A2M_h_NP_000005.2            TAYLVFSPSKSFVHLEPMSHELPCGHTQTVQAHYILNGGTLLGLKKLSFY 496
A2M_CM_XP_009001819.1        IAHLVFSPSKSFVHLEPIPHELPCGGQTQRIQAHYILKGDVLQGLKKFTFY 495
```

```
PZP_h_NP_002855.2          YLIMAKGVIVRSGTHTLPVESGDMKGSFALSFPVESDVAPIARMFIFAIL 542
PZP_CM_XP_009001820.1      YLIMAKGGIVRSGTHTLPVESGDMKGTFALSFPVESDIAPIARMFLFAIL 542
A2ML1_h_NP_001269353.1     -MLIGKGSLVMEGQKHLNSKKKGLKASFSLSLTFTSRLAPDPSLVIYAIF 49
A2ML1_CM_XP_009001807.1    YYLIGKGNLVMEGQKHLNPKKKGPKGSFSLSLIFTSRLAPDPSLVIYAIF 384
A2M_h_NP_000005.2          YLIMAKGGIVRTGTHGLLVKQEDMKGHFSISIPVKSDIAPVARLLIYAVL 546
A2M_CM_XP_009001819.1      SVIMAKGGIVHTGTYGLFVKEGDMKGHFSISIPVKSDFAPVARLLIYAIL 545

PZP_h_NP_002855.2          PDGEVVGDSEKFEIENCLANKVDLSFSPAQSPPASHAHLQVAAAPQSLCA 592
PZP_CM_XP_009001820.1      PDGEVVGDSEKFEIENCLANKVDLTFSPAQSLPASHAHLRVAAAPQSLCA 592
A2ML1_h_NP_001269353.1     PSGGVVADKIQFSVEMCFDNQVSLGFSPSQQLPGAEVELQLQAAPGSLCA 99
A2ML1_CM_XP_009001807.1    PSGGVVADKIQFSVEMCFDNQVSLGFSPTQQLPGAEVELQLQAAPRSLCA 434
A2M_h_NP_000005.2          PTGDVIGDSAKYDVENCLANKVDLSFSPSQSLPASHAHLRVTAAPQSVCA 596
A2M_CM_XP_009001819.1      PTGDVVGDSAKYKVENCLANKVDLSFRPSQSLPASQAHLQVTAAPQSVCA 595

PZP_h_NP_002855.2          LRAVDQSVLLMKPEAELSVSSVYNLLTVKD-----------LTNFPDNVD 631
PZP_CM_XP_009001820.1      LRAVDQSVLLMKPEAELSRSSVYNLLTVKD-----------LTSFPDNVN 631
A2ML1_h_NP_001269353.1     LRAVDESVLLLRPDRELSNRSVYGMFPFWYGHYPYQVAEYDQCPVSGPWD 149
A2ML1_CM_XP_009001807.1    LRAVDESVLLLRPETELSNHSVYGMFPFWYGRYPYQVAEY-MCPASGLWD 483
A2M_h_NP_000005.2          LRAVDQSVLLMKPDAELSASSVYNLLPEKD-----------LTGFPGPLN 635
A2M_CM_XP_009001819.1      LRAVDQSVLLMNTESELSTSSVYDLLPVKD-----------LTGFPEYLN 634

PZP_h_NP_002855.2          QQEEEQGHCPRPFFIHNGAIYVPLSSN-EADIYSFLKGMGLKVFTNSKIR 680
PZP_CM_XP_009001820.1      QQEEEQEPCPNSFIFRYGAFYVPLSRNDEADIYSFLKEMGLKVFTNSKVR 681
A2ML1_h_NP_001269353.1     FPQPLIDPMPQGHSSQRSIIWR-PSFSEGTDLFSFFRDVGLKILSNAKIK 198
A2ML1_CM_XP_009001807.1    FPQPLIDPVPQGHSSMRSAIWR-PWFSGGTDLFSFFQDVGLKILSNAKIK 532
A2M_h_NP_000005.2          DQDDE-DCINRHNVYINGITYTPVSSTNEKDMYSFLEDMGLKAFTNSKIR 684
A2M_CM_XP_009001819.1      DHDDG-DCINRHNIFVNGITYSPVSSTNEKDMYSFLRDMGLKALTNSKIH 683

PZP_h_NP_002855.2          KPKSCSVIPSVSAGAVGQGYYGAGLGVVERPYVPQLGTYNVIPLNNEQSS 730
PZP_CM_XP_009001820.1      KRKSCAVTHIAPAGGMSQGYYGASG-MAAMPYTPQFDAYNAIPLSNEQNS 730
A2ML1_h_NP_001269353.1     KPVDCSHRSPEYSTAMGAGGG-----------------HPEAFESSTPLH 231
A2ML1_CM_XP_009001807.1    KPVDCSHPSPNYGTPMAKVFSGDGD-------------NPRAFESSSSSH 569
A2M_h_NP_000005.2          KPKMCPQLQQYEMHGPEG---------LRVGFYESDVMGRGHARLVHVEE 725
A2M_CM_XP_009001819.1      KPNICSRPEPLAMYAPEG---------LRRAHYDSEVMLSGNALMMHVEE 724

PZP_h_NP_002855.2          GPVPETVRSYFPETWIWELVAVNSSGVAEVGVTVPDTITEWKAGAFCLSE 780
PZP_CM_XP_009001820.1      GPVPETVRSYFPETWIWELVAVDSSGVAEVGVTVPDTITEWKAGAFCLSE 780
A2ML1_h_NP_001269353.1     QAEDSQVRQYkFPETWLWDLFPIGNSGKEAVHVTVPDAITEWKAMSFCTSQ 281
A2ML1_CM_XP_009001807.1    RAEDSHVRQYFPETWLWDLFPIGNSGKEAIHVTVPDTITEWKAMSFCTSQ 619
A2M_h_NP_000005.2          P-HTETVRKYFPETWIWDLVVVNSAGVAEVGVTVPDTITEWKAGAFCLSE 774
A2M_CM_XP_009001819.1      PPHTETIRKYFPETWIWDLVVVNSAGEAEVEVTVPDTITEWKAGAFCLSK 774

PZP_h_NP_002855.2          DAGLGISSTASLRAFQPFFVELTMPYSVIRGEVFTLKATVLNYLPKCIRV 830
PZP_CM_XP_009001820.1      DAGLGISPTASLRAFQPFFVELTMPYSVIRGEVFTLKATVLNYLPKCIRV 830
A2ML1_h_NP_001269353.1     SRGFGLSPTVGLTAFKPFFVDLTLPYSVVRGESFRLTATIFNYLKDCIRV 331
A2ML1_CM_XP_009001807.1    SRGFGLSPTIGLTAFKPFFVDLTLPYSVVRGESFRLTATIFNYLKDCIRV 669
A2M_h_NP_000005.2          DAGLGISSTASLRAFQPFFVELTMPYSVIRGEAFTLKATVLNYLPKCIRV 824
A2M_CM_XP_009001819.1      DAGLGISPTASLQAFQPFFVELTMPYSVIRGEVFTLKATVLNYLPKCIRV 824

PZP_h_NP_002855.2          SVQLKASPAFLASQNTKGEESYCICGNERQTLSWTVTPKTLGNVNFSVSA 880
PZP_CM_XP_009001820.1      SVQLKASPAFLASKNSKGEESYCICGNERRTLSWTVTPKTLGNVNFSVSA 880
A2ML1_h_NP_001269353.1     QTDLAKSHEYQLESWADSQTSSCLCADEAKTHHWNITAVKLGHINFTIST 381
A2ML1_CM_XP_009001807.1    QTDLYKSPEYQLDSWADSQTSSCLCANEAKTYHWNITAVKLGHVNFTIST 719
A2M_h_NP_000005.2          SVQLEASPAFLAVPVEKEQAPHCICANGRQTVSWAVTPKSLGNVNFTVSA 874
A2M_CM_XP_009001819.1      SVQLEVSPAFLAVPVEKEQGPHCICVNGRQTVSWAITPKSLGNVNFTVSA 874

PZP_h_NP_002855.2          EAMQSLELCGNEVVEVPEIKRKDTVIKTLLVEAEGIEQEKTFSSMTCASG 930
PZP_CM_XP_009001820.1      EAMQSLELCGNEVAEVPEIKRKDTVIKTLLVEAEGIEKEKTFNSMTCASG 930
A2ML1_h_NP_001269353.1     KILDSNEPCGGQKGFVPQKGRSDTLIKPVLVKPEGVLVEKTHSSLLCPKG 431
A2ML1_CM_XP_009001807.1    KILDSNEPCGGQKGFVPQKGQSDMLIKPVLVKPEGVLVEKTHSSLLCPKG 769
A2M_h_NP_000005.2          EALESQELCGTEVPSVPEHGRKDTVIKPLLVEPEGLEKETTFNSLLCPSG 924
A2M_CM_XP_009001819.1      EALESQELCGTEVTSVPEYGKKDTIIKPLLVEPEGLEKETTFNSLLCPSG 924

PZP_h_NP_002855.2          ANVSEQLSLKLPSNVVKESARASFSVLGDILGSAMQNIQNLLQMPYGCGE 980
PZP_CM_XP_009001820.1      AEVSEQLSLKLPPNVVKESARASFSVLGDILGSAMQNIQNLLQMPYGCGE 980
A2ML1_h_NP_001269353.1     KVASESVSLELPVDIVPDSTKAYVTVLGDIMGTALQNLDGLVQMPSGCGE 481
A2ML1_CM_XP_009001807.1    KVVSESVSLEVPEDVVPDSTKAYVTVLGDIMGTALQNLDGLVQMPSGCGE 819
A2M_h_NP_000005.2          GEVSEELSLKLPPNVVEESARASVSVLGDILGSAMQNTQNLLQMPYGCGE 974
A2M_CM_XP_009001819.1      GEVSEELSLKLPPNVVEESARASVSVLGDILGSAMQNTQNLLQMPYGCGE 974

PZP_h_NP_002855.2          QNMVLFAPNIYVLNYLNETQQLTQEIKAKAVGYLITGYQRQLNYKHQDGS 1030
PZP_CM_XP_009001820.1      QNMVLFAPNIYVLSYLNETQQLTEEIKAKAIGYLISGYQRQLNYKHQDGS 1030
A2ML1_h_NP_001269353.1     QNMVLFAPIIYVLQYLEKAGLLTEEIRSRAVGFLEIGYQKELMYKHSNGS 531
A2ML1_CM_XP_009001807.1    QNMVLFAPIIYVLQYLEKAGLLTEKIKSRAVGFLEIGYQKELMYKHSNGS 869
A2M_h_NP_000005.2          QNMVLFAPNIYVLDYLNETQQLTPEIKSKAIGYLNTGYQRQLNYKHYDGS 1024
A2M_CM_XP_009001819.1      QNMVLFAPNIYVLDYLNKTQQLTPEIKSKAIGYLNTGYQRQLNYKHSDGS 1024
```

```
PZP_h_NP_002855.2           YSTFGERYGRNQGNTWLTAFVLKTFAQARSYIFIDEAHITQSLTWLSQMQ 1080
PZP_CM_XP_009001820.1       YSTFGEKYGWTQGNTWLTAFVLKTFSQARAYIFIDEVHITQSLTWLSQMQ 1080
A2ML1_h_NP_001269353.1      YSAFGERDG--NGNTWLTAFVTKCFGQAQKFIFIDPKNIQDALKWMAGNQ 579
A2ML1_CM_XP_009001807.1     YSAFGERDG--NGNTWLTAFVTKCFGQAQKFIFIDPKNIQDALKWMAGNQ 917
A2M_h_NP_000005.2           YSTFGERYGRNQGNTWLTAFVLKTFAQARAYIFIDEAHITQALIWLSQRQ 1074
A2M_CM_XP_009001819.1       YSTFGEQHGRNPGNTWLTAFVLKTFAQARAYIFIDEAHINQAFTWLSQKQ 1074

PZP_h_NP_002855.2           KDNGCFRSSGSLLNNAIKGGVEDEATLSAYVTIALLEIPLPVTNPIVRNA 1130
PZP_CM_XP_009001820.1       KDNGCFRSSGSLFNNAIKGGVEDEVTLSAYVTIALLEIPLPVTHPIVRNA 1130
A2ML1_h_NP_001269353.1      LPSGCYANVGNLLHTAMKGGVDDEVSLTAYVTAALLEMGKDVDDPMVSQG 629
A2ML1_CM_XP_009001807.1     LPSGCYANVGELFHTAMKGGVDDEVSLTAYVTAALLEMGKAIDDPMVSQG 967
A2M_h_NP_000005.2           KDNGCFRSSGSLLNNAIKGGVEDEVTLSAYITIALLEIPLTVTHPVVRNA 1124
A2M_CM_XP_009001819.1       KDTGCFRSSGSLLNNAIKGGVEDEVTLSAYVTIALLEIPIPVSNHVVRNA 1124

PZP_h_NP_002855.2           LFCLESAWNVAKEGTHGSHVYTKALLAYAFSLLGKQNQNREILNSLDKEA 1180
PZP_CM_XP_009001820.1       VFCLESAWNVAKEGTHGSQVYTKALLAYAFSLVGNQNRLREILNSLDAEA 1180
A2ML1_h_NP_001269353.1      LRCLKNSATSTTN------LYTQALLAYIFSLAGEMDIRNILLKQLDQQA 673
A2ML1_CM_XP_009001807.1     LQCLRNSVTSTTN------LYTQALLTYIFSLAGEMDVRNILLKQLDQQA 1011
A2M_h_NP_000005.2           LFCLESAWKTAQEGDHGSHVYTKALLAYAFALAGNQDKRKEVLKSLNEEA 1174
A2M_CM_XP_009001819.1       LFCLESAWKMAQEGASGSHVYTKALLAYAFALAGNQDKEKEVLESLNEEA 1174

PZP_h_NP_002855.2           VKEDNLVHWERPQRPKAPVGHLYQTQAPSAEVEMTSYVLLAYLTAQPAPT 1230
PZP_CM_XP_009001820.1       VKEDNLIHWERPQKPRAPVGHFYQPQAPSAEVEMTSYVLLAHLTAQPAPT 1230
A2ML1_h_NP_001269353.1      IISGESIYWS--QKPTPSSNASPWSEPAAVDVELTAYALLAQLTKP-SLT 720
A2ML1_CM_XP_009001807.1     IISGESIHWS--QKPTQSLNASPWSEPAALDVELTAYALLAQLTKP-SLT 1058
A2M_h_NP_000005.2           VKKDNSVHWERPQKPKAPVGHFYEPQAPSAEVEMTSYVLLAYLTAQPAPT 1224
A2M_CM_XP_009001819.1       VKKDNSVHWERPQKPKAPVGHFYQPQAPSAEVEMTSYVLLAHLTAQPAPS 1224

PZP_h_NP_002855.2           SGDLTSATNIVKWIMKQQNAQGGFSSTQDTVVALHALSRYGAATFTRTEK 1280
PZP_CM_XP_009001820.1       SRDLTTATNIVKWTMKQQNSQGGFSSTQDTVVALHALSRYGAITFTRTEK 1280
A2ML1_h_NP_001269353.1      QKEIAKATSIVAWLAKQRNAYGGFSSTQDTVVALQALAKYATTAYMPSEE 770
A2ML1_CM_XP_009001807.1     QKEIAKATSIVAWLAKQRNAYGGFSSTQDTVVALQALAKYATTAYMPSED 1108
A2M_h_NP_000005.2           SEDLTSATNIVKWITKQQNAQGGFSSTQDTVVALHALSKYGAATFTRTGK 1274
A2M_CM_XP_009001819.1       SEDLTAATNIVKWITKQQNSQGGFSSTQDTVVALHALSKYGAATFTKSGK 1274

PZP_h_NP_002855.2           TAQVTVQ-DSQTFSTNFQVDNNNLLLLQQISLPELPGEYVITVTGERCVY 1329
PZP_CM_XP_009001820.1       TAQVIIK-DSQTFSTNFQVDSNNLLLLQQTSLPELPGEYVITVTGERCVY 1329
A2ML1_h_NP_001269353.1      INLVVKS--TENFQRTFNIQSVNRLVFQQDTLPNVPGMYTLEASGQGCVY 818
A2ML1_CM_XP_009001807.1     ISLAVKS--TENFQRTFNIHSANRLVFQQETLPNVPGMYMLEASGQGCVY 1156
A2M_h_NP_000005.2           AAQVTIQ-SSGTFSSKFQVDNNNRLLLQQVSLPELPGEYSMKVTGEGCVY 1323
A2M_CM_XP_009001819.1       AAQVTVIQSSGTFSKNFQVDNDNRLLLQQVSLPQLPGEYSTKVTGEGCVY 1324

PZP_h_NP_002855.2           LQTSMKYNILPEKEDSPFALKVQTVPQTCDGHKAHTSFQISLTISYTGNR 1379
PZP_CM_XP_009001820.1       LQTSMKYNILPEKEDSPFALKVQTVPQTCDGHKAHTSFEISLTISYTGSR 1379
A2ML1_h_NP_001269353.1      VQTVLRYNILPPTNMKTFSLSVEIGKARCEQPTSPRSLTLTIHTSYVGSR 868
A2ML1_CM_XP_009001807.1     VQTVLKYNILPPKNMKTFTLSVEMGKARCEQLTSPRSLTLTIHTSYVGSR 1206
A2M_h_NP_000005.2           LQTSLKYNILPEKEEFPFALGVQTLPQTCDEPKAHTSFQISLSVSYTGSR 1373
A2M_CM_XP_009001819.1       LQTSLKYSILPEKEEFPFALEVHTLPQTCDEPKARTSFEISLNVSYTGSR 1374

PZP_h_NP_002855.2           PASNMVIVDVKMVSGFIPLKPTVKMLERSSSVSRTEVSNNHVLIYVEQV- 1428
PZP_CM_XP_009001820.1       PASNMVIVDVKMVSGFIPLKPTVKMLERSSSVSRTEVSNNHVLIYVEQVR 1429
A2ML1_h_NP_001269353.1      SSSNMAIVEVKMLSGFSPMEGTNQLLLQQPLVKKVEFGTDTLNIYLDELI 918
A2ML1_CM_XP_009001807.1     SSSNMAIVEVKMLSGFNPMEGTSQSLLQQPLVKKVEFGTDTLNIYLIELT 1256
A2M_h_NP_000005.2           SASNMAIVDVKMVSGFIPLKPTVKMLERSNHVSRTEVSSNHVLIYLDKVS 1423
A2M_CM_XP_009001819.1       SASNMAIADVKMISGFIPLKPTVKMLERSNHVSRTEVNNNHVLIYLDKVS 1424

PZP_h_NP_002855.2           --------TNQTLSFSFMVLQDIPVGDLKPAIVKVYDYYETDESVVAEYI 1470
PZP_CM_XP_009001820.1       ALTSWLRVTNQTLSFSFTVLQDVPVRDLKPAIVKVYDYYETDESVVAEYI 1479
A2ML1_h_NP_001269353.1      KN---------TQTYTFTISQSVLVTNLKPATIKVYDYYLPDEQATIQYS 959
A2ML1_CM_XP_009001807.1     KN---------TQTYTFTISQSVLVTNLKPAAIKVYDYYLPDEQATIQYS 1297
A2M_h_NP_000005.2           ---------NQTLSLFFTVLQDVPVRDLKPAIVKVYDYYETDEFAIAEYN 1464
A2M_CM_XP_009001819.1       ---------NQTLSLSFTVLQDVPVRDLKPAIVKVYDYYETDEFAIAEYN 1465

PZP_h_NP_002855.2           APCSTDTEHGNV 1482
PZP_CM_XP_009001820.1       APCSTDTEHGNV 1491
A2ML1_h_NP_001269353.1      DPCE-------- 963
A2ML1_CM_XP_009001807.1     DPCE-------- 1301
A2M_h_NP_000005.2           APCSKDLGNA-- 1474
A2M_CM_XP_009001819.1       APCSKDLGNA-1475
```

Supplemental Fig.3 Alignment of A2M family proteins.
ClustalW2 was used for protein structure alignment. Grey: predicted bait region;
Yellow: disulfide moiety; Green: thiol-ester site; Pink: cutting site (CS) of PE; Blue: CS of Asp-N E;
Red: CS of LysC; Light blue: LysN.

Supplemental Fig.4

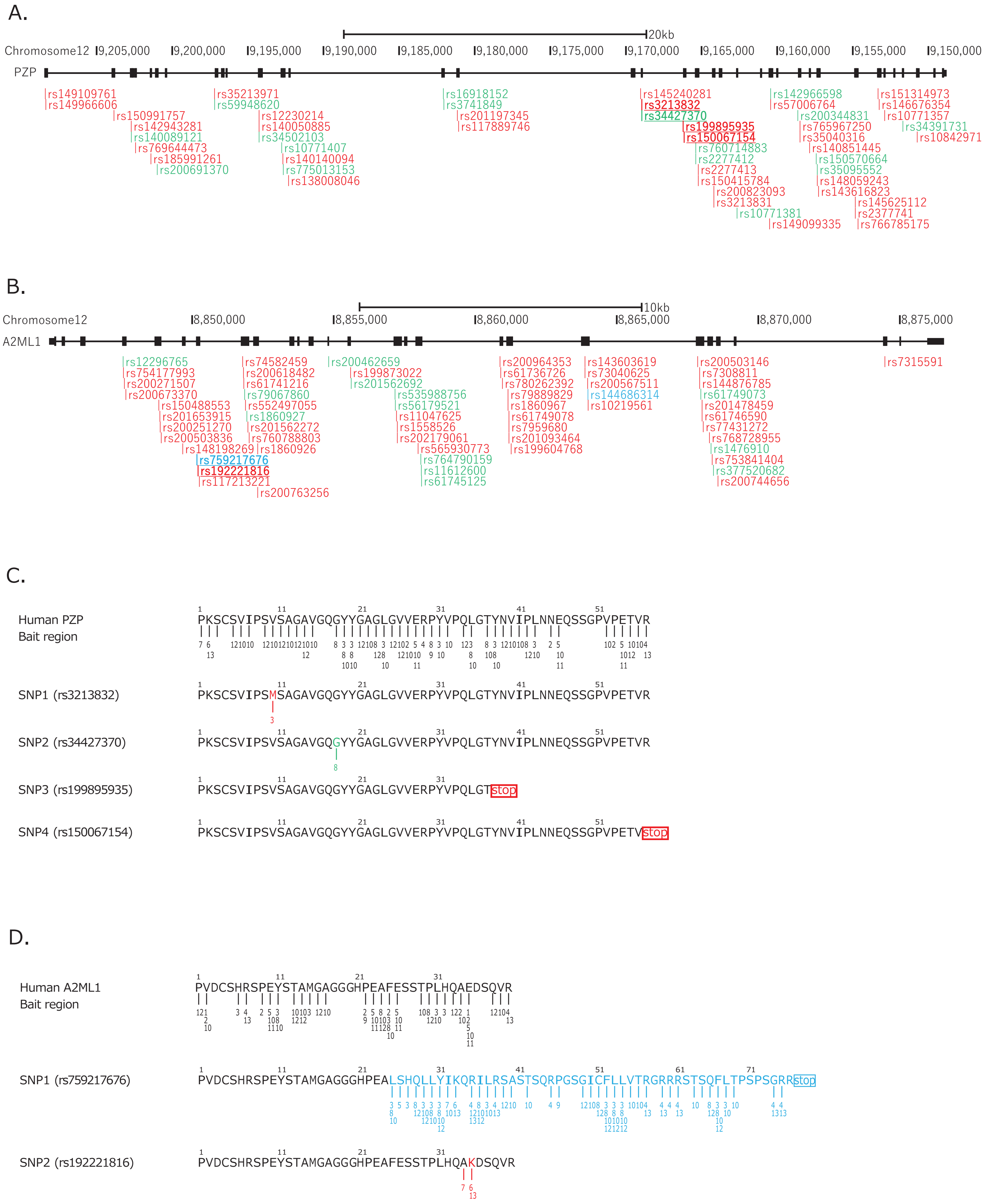

Supplemental Fig. 4. Evolutional characteristics of human PZP and A2ML1.
A. Single nucleotide polymorphisms (SNPs) in human PZP; B. SNPs in human A2ML1;
C. Protease cutting sites in the bait region of human PZP; D. Protease cutting sites in bait region of human A2ML1.
Panels A and B: UCSC Genome Browser (https://genome.ucsc.edu/) was used for analysis of SNPs.
Only SNPs for which MAF was ≥1% were included.
rs: reference SNP ID number; red: SNPs with amino acid substitutions (missense mutations, nonsense mutations);
green: synonymous mutations; blue: frameshift mutations; underlined rs IDs: SNPs in bait region.
Panels C and D: PeptideCutter analysis of protease cutting sites. Changes to cutting sites in each SNP are shown.
Positions of cuttings sites are indicated by the accompanying numerals: 1: Aspartic acid-N endopeptidase;
2: aspartic acid-N endopeptidase + N-terminal glutamic acid; 3: chymotrypsin; 4: clostripain;
5: glutamyl endopeptidase; 6: lysyl endopeptidase C; 7: peptidyl-Lys metalloendopeptidase; 8: pepsin;
9: prolyl-endopeptidase; 10: proteinase K; 11: staphylococcal peptidase; 12: thermolysin; 13: trypsin;
stop: stop codon.

Supplemental Fig.5

A.

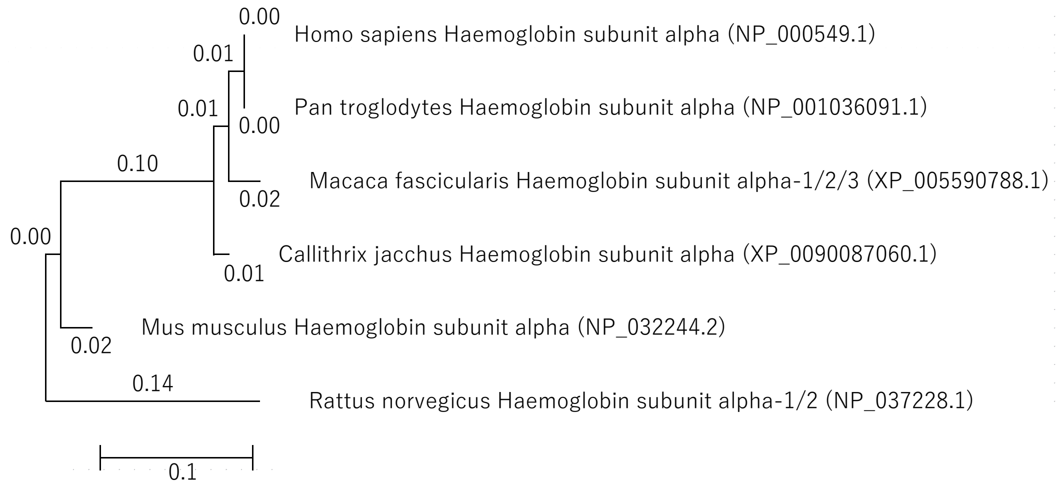

B.

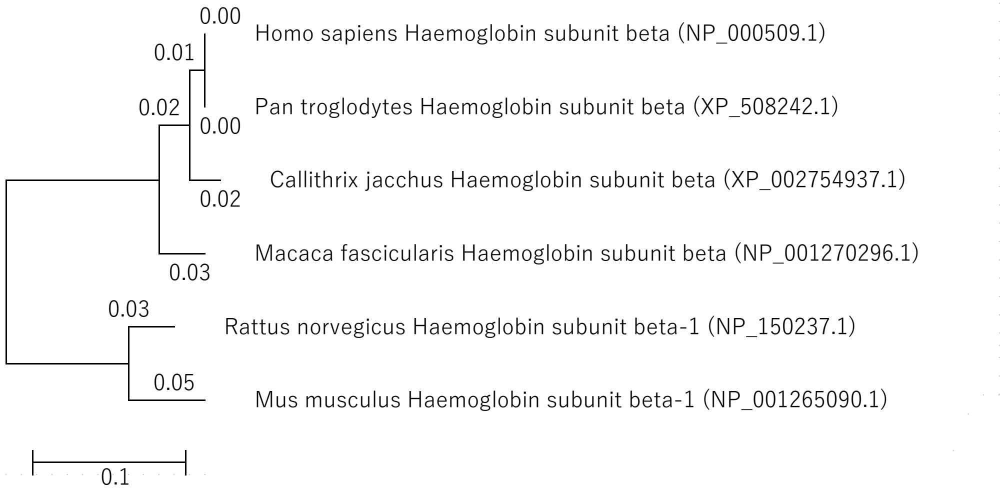

Supplemental Fig. 5. Phylogeny of haemoglobin α and β proteins from primates and rodents inferred using MEGA6.06.
A: Haemoglobin subunit α sequence; B: Haemoglobin subunit β sequence.
NCBI sequence accession numbers are indicated. Numbers at branches indicate bootstrap values.
Branch lengths represent the amount of change estimated to have occurred between nodes.

Supplemental Table 1

A. Human

Category		No.	Identified Proteins	UniProt EntryName	UniProt Accession No.	Molecular Weight	Average 1st	2nd	3rd	ratio 2nd/1st	ratio 3rd/1st	1st trimester H1	H2	H3	2nd trimester H1	H2	H3	3rd trimester H1	H2	H3
1	1a	1	Pregnancy zone protein	PZP_HUMAN	P20742	164 kDa	2.77	11.32	7.25	4.08	2.61	8.32	0.00	0.00	25.59	4.32	4.03	21.75	0.00	0.00
		2	Antithrombin-III	ANT3_HUMAN	P01008	53 kDa	0.33	2.02	0.31	6.07	0.92	0.00	0.00	1.00	0.00	6.05	0.00	0.00	0.00	0.92
		3	Alpha-1-antichymotrypsin	AACT_HUMAN	P01011	48 kDa	0.00	2.02	0.00			0.00	0.00	0.00	0.00	6.05	0.00	0.00	0.00	0.00
		4	Complement C1q subcomponent subunit C	C1QC_HUMAN	P02747	26 kDa	0.00	1.15	0.00			0.00	0.00	0.00	0.00	3.46	0.00	0.00	0.00	0.00
	1b	5	Serotransferrin	TRFE_HUMAN	P02787	77 kDa	34.12	52.12	97.23	1.53	2.85	39.51	30.93	31.92	29.68	69.20	57.49	86.98	26.89	177.83
		6	Apolipoprotein C-II	APOC2_HUMAN	P02655	11 kDa	10.65	12.57	23.27	1.18	2.18	5.20	12.80	13.96	7.16	10.38	20.17	20.71	28.01	21.08
		7	Sex hormone-binding globulin	SHBG_HUMAN	P04278	44 kDa	9.52	15.90	23.72	1.67	2.49	13.52	1.07	13.96	20.47	6.05	21.18	28.99	20.17	22.00
		8	Fibronectin	FINC_HUMAN	P02751	263 kDa	4.82	6.10	15.77	1.27	3.27	0.00	7.47	6.98	0.00	17.30	1.01	15.53	13.45	18.33
		9	Apolipoprotein L1	APOL1_HUMAN	O14791	44 kDa	3.09	2.55	6.96	0.83	2.25	2.08	3.20	3.99	1.02	2.60	4.03	6.21	10.08	4.58
		10	Hemoglobin subunit beta	HBB_HUMAN	P68871	16 kDa	0.36	0.97	4.40	2.73	12.37	0.00	1.07	0.00	2.05	0.86	0.00	3.11	0.00	10.08
		11	Hemoglobin subunit alpha	HBA_HUMAN	P69905	15 kDa	0.00	1.65	3.75			0.00	0.00	0.00	3.07	0.86	1.01	2.07	0.00	9.17
		12	Fibulin-1	FBLN1_HUMAN	P23142	77 kDa	0.00	0.91	6.95			0.00	0.00	0.00	0.00	1.73	1.01	5.18	5.60	10.08
		13	Complement component C8 alpha chain	CO8A_HUMAN	P07357	65 kDa	0.00	0.29	1.02			0.00	0.00	0.00	0.00	0.86	0.00	1.04	1.12	0.92
		14	Pregnancy-specific beta-1-glycoprotein 1	PSG1_HUMAN	P11464	47 kDa	0.00	0.00	18.32			0.00	0.00	0.00	0.00	0.00	0.00	21.75	11.20	22.00
		15	Actin, cytoplasmic 1	ACTB_HUMAN	P60709	42 kDa	0.00	0.00	7.33			0.00	0.00	0.00	0.00	0.00	0.00	0.00	0.00	22.00
		16	Pregnancy-specific beta-1-glycoprotein 9	PSG9_HUMAN	Q00887	48 kDa	0.00	0.00	4.94			0.00	0.00	0.00	0.00	0.00	0.00	9.32	0.00	5.50
2	2a	17	Complement C5	CO5_HUMAN	P01031	188 kDa	10.65	4.28	5.49	0.40	0.52	8.32	10.67	12.97	1.02	1.73	10.09	5.18	6.72	4.58
		18	Complement C1s subcomponent	C1S_HUMAN	P09871	77 kDa	2.76	1.01	1.77	0.37	0.64	2.08	3.20	2.99	0.00	0.00	3.03	1.04	3.36	0.92
		19	Plasma kallikrein	KLKB1_HUMAN	P03952	71 kDa	2.35	0.96	1.29	0.41	0.55	0.00	1.07	5.98	0.00	0.86	2.02	0.00	1.12	2.75
		20	Carboxypeptidase N subunit 2	CPN2_HUMAN	P22792	61 kDa	1.34	0.00	1.53		1.14	1.04	0.00	2.99	0.00	0.00	0.00	0.00	0.00	4.58
	2b	21	Ig gamma-1 chain C region	IGHG1_HUMAN	P01857	36 kDa	24.73	20.00	10.54	0.81	0.43	29.12	18.13	26.93	22.52	23.36	14.12	9.32	6.72	15.58
		22	Ig lambda-2 chain C regions	LAC2_HUMAN	P0CG05	11 kDa	9.42	11.09	4.37	1.18	0.46	7.28	16.00	4.99	7.16	19.03	7.06	4.14	8.96	0.00
		23	Ig kappa chain C region	IGKC_HUMAN	P01834	12 kDa	2.76	3.14	1.00	1.14	0.36	2.08	3.20	2.99	3.07	4.32	2.02	2.07	0.00	0.92
		24	Histidine-rich glycoprotein	HRG_HUMAN	P04196	60 kDa	1.76	0.97	0.00	0.55		2.08	3.20	0.00	2.05	0.86	0.00	0.00	0.00	0.00
		25	Inter-alpha-trypsin inhibitor heavy chain H3	ITIH3_HUMAN	Q06033	100 kDa	1.40	0.68	2.59	0.49	1.84	2.08	2.13	0.00	2.05	0.00	0.00	1.04	6.72	0.00
		26	Leucine-rich alpha-2-glycoprotein	A2GL_HUMAN	P02750	38 kDa	0.68	1.25	0.31	1.85	0.45	1.04	0.00	1.00	1.02	1.73	1.01	0.00	0.00	0.92
3		27	Serum albumin	ALBU_HUMAN	P02768	69 kDa	1227.97	1233.13	829.13	1.00	0.68	1384.00	1044.10	1255.80	1478.90	1217.90	1002.60	960.93	676.75	849.72
		28	Ceruloplasmin	CERU_HUMAN	P00450	122 kDa	385.27	394.02	407.61	1.02	1.06	372.26	393.55	390.01	390.97	332.16	458.94	401.77	477.31	343.74
		29	Fibrinogen alpha chain	FIBA_HUMAN	P02671	95 kDa	353.15	273.27	377.39	0.77	1.07	330.67	394.62	334.15	223.12	300.15	296.54	339.64	451.54	340.99
		30	Fibrinogen beta chain	FIBB_HUMAN	P02675	56 kDa	289.56	241.50	335.76	0.83	1.16	267.24	292.23	309.21	200.60	270.74	253.17	271.30	347.34	388.65
		31	Complement C3	CO3_HUMAN	P01024	187 kDa	271.35	250.21	312.39	0.92	1.15	186.13	300.76	327.17	185.25	191.16	374.21	261.98	350.70	324.49
		32	Fibrinogen gamma chain	FIBG_HUMAN	P02679	52 kDa	214.79	188.37	240.05	0.88	1.12	193.41	232.51	218.44	149.43	236.14	179.54	225.74	248.74	245.66
		33	Alpha-1-antitrypsin	A1AT_HUMAN	P01009	47 kDa	205.74	308.35	268.10	1.50	1.30	203.81	161.05	252.36	276.34	266.42	382.28	302.36	209.52	292.41
		34	Haptoglobin	HPT_HUMAN	P00738	45 kDa	172.57	141.81	104.00	0.82	0.60	189.25	235.71	92.76	137.15	207.60	80.69	124.26	140.06	47.67
		35	Hemopexin	HEMO_HUMAN	P02790	52 kDa	152.66	136.41	163.55	0.89	1.07	141.42	174.91	141.64	136.12	122.83	150.29	131.51	190.48	168.66
		36	Apolipoprotein A-I	APOA1_HUMAN	P02647	31 kDa	136.40	213.09	169.99	1.56	1.25	133.10	135.45	140.64	246.66	180.78	211.82	166.71	173.67	169.58
		37	Alpha-2-HS-glycoprotein	FETUA_HUMAN	P02765	39 kDa	131.85	124.58	142.81	0.94	1.08	126.86	129.05	139.64	123.84	110.72	139.19	144.97	132.21	151.24
		38	Inter-alpha-trypsin inhibitor heavy chain H2	ITIH2_HUMAN	P19823	106 kDa	110.25	93.59	120.64	0.85	1.09	120.62	133.32	76.80	110.54	86.50	83.72	127.36	155.74	78.83
		39	Inter-alpha-trypsin inhibitor heavy chain H1	ITIH1_HUMAN	P19827	101 kDa	83.69	73.29	89.62	0.88	1.07	88.39	93.86	68.83	85.97	74.39	59.51	97.34	116.53	55.00
		40	Apolipoprotein A-II	APOA2_HUMAN	P02652	11 kDa	78.18	78.04	73.67	1.00	0.94	76.95	76.79	80.79	85.97	67.47	80.69	73.52	75.07	72.41
		41	Complement factor B	CFAB_HUMAN	P00751	86 kDa	67.99	68.46	67.06	1.01	0.99	69.67	102.39	31.92	69.60	87.36	48.42	81.80	91.88	27.50
		42	Haptoglobin-related protein	HPTR_HUMAN	P00739	39 kDa	67.08	54.02	45.82	0.81	0.68	71.75	89.59	39.90	53.22	73.52	35.30	59.02	58.26	20.17
		43	Prothrombin	THRB_HUMAN	P00734	70 kDa	64.24	56.42	51.84	0.88	0.81	68.63	52.26	71.82	63.46	49.30	56.49	54.88	39.22	61.42
		44	Inter-alpha-trypsin inhibitor heavy chain H4	ITIH4_HUMAN	Q14624	103 kDa	61.62	54.75	37.47	0.89	0.61	66.55	55.46	62.84	56.29	72.66	35.30	47.63	33.61	31.17
		45	Vitamin D-binding protein	VTDB_HUMAN	P02774	53 kDa	57.50	67.16	58.53	1.17	1.02	57.19	55.46	59.85	69.60	68.33	63.55	55.92	58.26	61.42
		46	Plasminogen	PLMN_HUMAN	P00747	91 kDa	56.04	46.38	47.94	0.83	0.86	54.07	51.19	62.84	44.01	40.66	54.47	44.53	36.98	62.33
		47	Alpha-2-macroglobulin	A2MG_HUMAN	P01023	163 kDa	52.27	51.50	45.06	0.99	0.86	64.47	39.46	52.87	69.60	41.52	43.37	56.95	36.98	41.25
		48	Kininogen-1	KNG1_HUMAN	P01042	72 kDa	51.01	54.15	67.62	1.06	1.33	50.95	51.19	50.87	51.17	52.76	58.50	67.31	64.99	70.58
		49	Angiotensinogen	ANGT_HUMAN	P01019	53 kDa	42.29	47.92	54.72	1.13	1.29	38.47	40.53	47.88	33.78	72.66	37.32	53.85	47.06	63.25
		50	Alpha-1B-glycoprotein	A1BG_HUMAN	P04217	54 kDa	38.68	45.59	36.76	1.18	0.95	41.59	40.53	33.91	49.13	49.30	38.33	35.21	34.73	40.33
		51	Clusterin	CLUS_HUMAN	P10909	52 kDa	35.19	27.95	29.83	0.79	0.85	36.40	36.26	32.92	28.66	25.95	29.25	27.96	35.85	25.67
		52	Apolipoprotein B-100	APOB_HUMAN	P04114	516 kDa	33.03	49.32	60.69	1.49	1.84	14.56	26.66	57.85	27.63	40.66	79.68	40.38	58.26	83.41
		53	Complement C4-A	CO4A_HUMAN	P0C0L4	193 kDa	32.12	36.91	23.77	1.15	0.74	29.12	37.33	29.92	22.52	57.95	30.26	24.85	33.61	12.83
		54	Vitronectin	VTNC_HUMAN	P04004	54 kDa	29.92	35.04	40.02	1.17	1.34	27.04	28.80	33.91	34.80	19.90	50.43	42.46	33.61	44.00
		55	Apolipoprotein A-IV	APOA4_HUMAN	P06727	45 kDa	29.48	31.56	55.49	1.07	1.88	35.36	34.13	18.95	29.68	33.74	31.27	59.02	51.54	55.92
		56	Ig gamma-3 chain C region	IGHG3_HUMAN	P01860	41 kDa	28.07	28.30	20.59	1.01	0.73	29.12	19.20	35.91	30.70	19.90	34.29	21.75	13.45	26.58
		57	Complement factor H	CFAH_HUMAN	P08603	139 kDa	26.45	30.30	31.55	1.15	1.19	24.96	23.46	30.92	27.63	25.95	37.32	26.92	24.65	43.08
		58	Serum paraoxonase/arylesterase 1	PON1_HUMAN	P27169	40 kDa	24.70	21.78	32.11	0.88	1.30	20.80	37.33	15.96	13.31	32.87	19.16	30.03	47.06	19.25
		59	Apolipoprotein C-III	APOC3_HUMAN	P02656	11 kDa	20.38	22.78	32.96	1.12	1.62	20.80	22.40	17.95	21.49	21.63	25.22	28.99	31.37	38.50
		60	Apolipoprotein E	APOE_HUMAN	P02649	36 kDa	19.81	25.85	39.09	1.30	1.97	19.76	11.73	27.93	20.47	14.71	42.36	20.71	36.98	59.58
		61	Beta-2-glycoprotein 1	APOH_HUMAN	P02749	38 kDa	17.91	19.50	18.96	1.09	1.06	18.72	17.07	17.95	19.45	13.84	25.22	21.75	16.81	18.33
		62	Ig alpha-1 chain C region	IGHA1_HUMAN	P01876	38 kDa	16.02	15.96	13.95	1.00	0.87	20.80	5.33	21.94	21.49	11.25	15.13	12.43	5.60	23.83
		63	Ig mu chain C region	IGHM_HUMAN	P01871	49 kDa	15.97	14.01	19.10	0.88	1.20	15.60	21.33	10.97	15.35	15.57	11.10	22.78	23.53	11.00
		64	Transthyretin	TTHY_HUMAN	P02766	16 kDa	15.82	27.56	17.11	1.74	1.08	18.72	12.80	15.96	37.87	21.63	23.20	17.60	19.05	14.67
		65	Complement component C9	CO9_HUMAN	P02748	63 kDa	13.22	13.33	17.90	1.01	1.35	7.28	22.40	9.97	5.12	14.71	20.17	14.50	29.13	10.08
		66	Apolipoprotein C-I	APOC1_HUMAN	P02654	9 kDa	12.80	12.80	17.11	1.00	1.34	14.56	13.87	9.97	5.12	18.17	15.13	18.64	13.45	19.25
		67	Ig gamma-2 chain C region	IGHG2_HUMAN	P01859	36 kDa	12.56	9.99	7.84	0.80	0.62	11.44	20.26	5.98	9.21	20.76	0.00	5.18	10.08	8.25
		68	C4b-binding protein alpha chain	C4BPA_HUMAN	P04003	67 kDa	11.01	11.23	11.88	1.02	1.08	7.28	12.80	12.97	8.19	10.38	15.13	11.39	12.33	11.92
		69	Serum amyloid A-4 protein	SAA4_HUMAN	P35542	15 kDa	8.94	9.87	8.36	1.10	0.94	8.32	8.53	9.97	5.12	10.38	14.12	8.28	6.72	10.08
		70	Afamin	AFAM_HUMAN	P43652	69 kDa	8.89	7.13	11.74	0.80	1.32	6.24	7.47	12.97	4.09	5.19	12.10	11.39	10.08	13.75
		71	Gelsolin	GELS_HUMAN	P06396	86 kDa	7.18	7.86	4.22	1.09	0.59	4.16	6.40	10.97	5.12	10.38	8.07	7.25	4.48	0.92
		72	Heparin cofactor 2	HEP2_HUMAN	P05546	57 kDa	6.85	7.87	9.51	1.15	1.39	8.32	4.27	7.98	7.16	10.38	6.05	13.46	2.24	12.83
		73	Protein AMBP	AMBP_HUMAN	P02760	39 kDa	6.19	7.97	4.28	1.29	0.69	8.32	4.27	5.98	9.21	8.65	6.05	6.21	1.12	5.50
		74	Pigment epithelium-derived factor	PEDF_HUMAN	P36955	46 kDa	5.17	4.48	5.80	0.87	1.12	5.20	5.33	4.99	3.07	4.32	6.05	5.18	6.72	5.50
		75	Complement factor I	CFAI_HUMAN	P05156	66 kDa	3.87	4.82	4.26	1.25	1.10	2.08	8.53	1.00	4.09	4.32	6.05	3.11	7.84	1.83
		76	Coagulation factor XII	FA12_HUMAN	P00748	68 kDa	2.41	3.57	3.62	1.48	1.50	1.04	3.20	2.99	4.09	2.60	4.03	4.14	6.72	0.00
		77	CD5 antigen-like	CD5L_HUMAN	O43866	38 kDa	1.75	1.94	1.67	1.11	0.96	3.12	2.13	0.00	4.09	1.73	0.00	2.07	1.12	1.83
		78	Alpha-2-antiplasmin	A2AP_HUMAN	P08697	55 kDa	1.70	2.59	1.74	1.52	1.02	3.12	0.00	1.99	0.00	7.78	0.00	2.07	2.24	0.92
		79	Complement component C6	CO6_HUMAN	P13671	105 kDa	0.33	0.67	0.35	2.02	1.04	0.00	0.00	1.00	0.00	0.00	2.02	1.04	0.00	0.00
		80	Complement component C8 beta chain	CO8B_HUMAN	P07358	67 kDa	0.00	0.58	0.00			0.00	0.00	0.00	0.00	1.73	0.00	0.00	0.00	0.00
		81	Corticosteroid-binding globulin	CBG_HUMAN	P08185	45 kDa	0.00	0.58	0.00			0.00	0.00	0.00	0.00	1.73	0.00	0.00	0.00	0.00
		82	Complement C1r subcomponent	C1R_HUMAN	P00736	80 kDa	0.00	0.34	0.00			0.00	0.00	0.00	0.00	0.00	1.01	0.00	0.00	0.00
		83	Platelet basic protein	CXCL7_HUMAN	P02775	14 kDa	0.00	0.00	0.31			0.00	0.00	0.00	0.00	0.00	0.00	0.00	0.00	0.92

B. Marmoset

Category		No.	Identified Proteins	NCBI GI No.	Accession No.	Molecular Weight	Average 1st	2nd	3rd	ratio 2nd/1st	ratio 3rd/1st	1st trimester M1	M2	M3	2nd trimester M1	M2	M3	3rd trimester M1	M2
1	1a	1	Alpha-2-macroglobulin-like	675677766	XP_002752342	165 kDa	15.07	259.86	55.58	17.25	3.69	31.05	0.00	14.15	331.99	217.41	230.18	75.30	35.86
		2	Hemoglobin alpha chain	122366	P18972*	15 kDa	9.41	128.91	27.39	13.69	2.91	10.09	9.06	9.10	120.17	138.98	127.57	7.44	47.34
		3	Hemoglobin beta chain	122577	P18985*	16 kDa	4.72	74.62	20.26	15.79	4.29	2.33	6.79	5.05	72.83	58.57	92.44	4.65	35.86
		4	Apolipoprotein A-II	296229454	XP_002760286	11 kDa	1.53	5.03	4.52	3.29	2.96	1.55	0.00	3.03	1.82	4.96	8.32	1.86	7.17
		5	Complement component C6	390460019	XP_002745070	106 kDa	0.52	4.27	1.39	8.26	2.70	1.55	0.00	0.00	6.07	3.97	2.77	2.79	0.00
		6	Pigment epithelium-derived factor	675657740	XP_008994618	47 kDa	0.97	4.63	0.93	4.76	0.96	0.78	1.13	1.01	0.00	13.90	0.00	1.86	0.00
		7	Immunoglobulin heavy chain variable region	20502564	AAM22526**	15 kDa	0.52	1.99	0.46	3.85	0.90	1.55	0.00	0.00	1.21	1.99	2.77	0.93	0.00
		8	Zinc-alpha-2-glycoprotein-like	675642469	XP_008981062	27 kDa	0.26	1.94	0.46	7.48	1.80	0.78	0.00	0.00	3.03	0.00	2.77	0.93	0.00
		9	Insulin-like growth factor-binding protein	296219288	XP_002755809	66 kDa	0.52	1.34	0.46	2.59	0.90	1.55	0.00	0.00	3.03	0.99	0.00	0.93	0.00
		10	Apolipoprotein E	296234066	XP_002762273	36 kDa	0.00	1.73	0.00			0.00	0.00	0.00	2.43	0.00	2.77	0.00	0.00
		11	Complement component C8 beta chain	296208073	XP_002750920	67 kDa	0.00	1.04	0.46			0.00	0.00	0.00	1.21	0.99	0.92	0.93	0.00
	1b	12	Plasma serine protease inhibitor	675692410	XP_002754293	50 kDa	0.93	2.25	2.87	2.41	3.08	0.78	0.00	2.02	0.00	3.97	2.77	0.00	5.74
2	2a	13	Haptoglobin	296231486	XP_002761163	38 kDa	71.36	0.00	10.69	0.00	0.15	87.70	19.24	107.14	0.00	0.00	0.00	21.38	0.00
	2b	14	Angiotensinogen	675750692	XP_008984059	54 kDa	29.15	22.65	11.49	0.78	0.39	32.60	21.51	33.35	17.60	19.86	30.51	15.80	7.17
		15	Alpha-1-acid glycoprotein 1	296229919	XP_002760492	25 kDa	14.29	6.26	3.29	0.44	0.23	16.30	21.51	5.05	6.07	9.93	2.77	3.72	2.87
		16	Complement C4-A	675651995	XP_002746418	193 kDa	14.26	14.11	4.65	0.99	0.33	20.96	5.66	16.17	12.14	8.93	21.26	9.30	0.00
		17	Gelsolin	675639222	XP_009000537	86 kDa	8.00	4.69	3.04	0.59	0.38	7.76	10.19	6.06	5.46	3.97	4.62	4.65	1.43
		18	Leucine-rich alpha-2-glycoprotein	296232579	XP_002761694	38 kDa	7.58	5.94	0.00	0.78		5.43	1.13	16.17	1.21	12.91	3.70	0.00	0.00
		19	Complement factor H	675747009	XP_008983383	140 kDa	5.10	4.57	1.86	0.90	0.36	6.99	2.26	6.06	7.89	3.97	1.85	3.72	0.00
		20	Serum amyloid A-4 protein	675698869	XP_009006030	15 kDa	4.63	2.46	1.39	0.53	0.30	5.43	3.40	5.05	1.82	0.00	5.55	2.79	0.00
		21	Plasminogen	675655078	XP_008993588	15 kDa	3.62	5.60	0.00	1.55		10.87	0.00	0.00	10.32	0.00	6.47	0.00	0.00
		22	Aminopeptidase N	675663052	XP_008996656	109 kDa	1.67	0.61	0.00	0.36		3.88	1.13	0.00	1.82	0.00	0.00	0.00	0.00
		23	Immunoglobulin heavy chain	20502592	AAM22540**	11 kDa	1.03	1.01	0.00	0.98		3.10	0.00	0.00	3.03	0.00	0.00	0.00	0.00
		24	Complement component C9	675646263	XP_002745089	65 kDa	1.03	0.81	0.00	0.78		3.10	0.00	0.00	2.43	0.00	0.00	0.00	0.00
3		25	Serum albumin	296196349	XP_002745783	69 kDa	2136.17	1753.00	2138.75	0.82	1.00	2010.90	2423.60	1974.00	1770.40	1877.30	1611.30	2084.20	2193.30
		26	Fibrinogen alpha chain	675647465	XP_008990825	74 kDa	207.23	189.92	231.01	0.92	1.11	197.13	189.05	235.50	159.62	210.46	199.67	229.62	232.39
		27	Fibrinogen beta chain	296195360	XP_002745405	56 kDa	183.31	144.74	198.52	0.79	1.08	194.03	142.63	213.26	107.43	140.97	185.81	177.56	219.48
		28	Hemopexin	390470175	XP_003734248	51 kDa	111.32	86.04	115.99	0.77	1.04	104.00	108.67	121.29	84.36	93.32	80.42	114.34	117.63
		29	Apolipoprotein A-I	296216259	XP_002754495	30 kDa	104.32	96.68	145.69	0.93	1.40	86.93	138.11	87.93	79.51	131.04	79.50	110.63	180.75
		30	Fibrinogen gamma chain	675647901	XP_008990984	53 kDa	84.48	60.22	75.66	0.71	0.90	81.49	86.03	85.91	45.52	69.49	65.63	75.30	76.03
		31	Inter-alpha-trypsin inhibitor heavy chain H4	675728479	XP_008980055	102 kDa	81.44	90.40	74.19	1.11	0.91	71.40	66.79	106.13	80.72	81.41	109.08	78.09	70.29
		32	Complement C3	675757099	XP_008985329	189 kDa	74.56	76.24	73.35	1.02	0.98	101.67	63.39	58.62	90.43	61.55	76.73	86.46	60.25
		33	Alpha-2-HS-glycoprotein	675726559	XP_008979714	46 kDa	68.75	57.56	63.96	0.84	0.93	72.96	55.47	77.83	48.56	57.58	66.56	57.64	70.29
		34	Ceruloplasmin	675738882	XP_008981931	122 kDa	61.20	68.69	57.08	1.12	0.93	41.91	67.92	73.78	55.84	60.56	89.67	53.92	60.25
		35	Alpha-2-macroglobulin	675678022	XP_009001819	163 kDa	55.61	67.17	62.71	1.21	1.13	65.97	45.28	55.59	75.26	61.55	64.71	78.09	47.34
		36	Inter-alpha-trypsin inhibitor heavy chain H2	296206127	XP_002750076	106 kDa	43.44	31.92	38.93	0.73	0.90	38.03	40.75	51.55	24.88	35.74	35.13	36.26	41.60
		37	Inter-alpha-trypsin inhibitor heavy chain H3	675728476	XP_008980054	100 kDa	41.78	37.10	35.30	0.89	0.84	40.36	38.49	46.49	35.81	35.74	39.75	39.04	31.56
		38	Alpha-1-antitrypsin	675692376	XP_003734117	47 kDa	34.39	31.62	19.93	0.92	0.58	35.70	26.04	41.44	33.99	24.82	36.05	26.96	12.91
		39	Alpha-1-antitrypsin precursor-like protein	146332359	ABQ22685**	24 kDa	28.41	32.14	29.82	1.13	1.05	28.72	18.11	38.41	36.42	25.81	34.20	38.12	21.52
		40	Vitronectin	296202224	XP_002748402	52 kDa	24.49	22.08	29.30	0.90	1.20	24.06	27.17	22.24	20.03	26.80	19.41	24.17	34.43
		41	Serum paraoxonase/arylesterase 1	296209662	XP_002751634	40 kDa	24.37	18.55	25.16	0.76	1.03	20.96	33.96	18.19	10.93	31.77	12.94	13.02	37.30
		42	Prothrombin	675700193	XP_003734294	70 kDa	23.73	22.56	25.17	0.95	1.06	24.06	24.90	22.24	22.46	25.81	19.41	28.82	21.52
		43	Inter-alpha-trypsin inhibitor heavy chain H1	675728748	XP_008980090	103 kDa	20.23	16.79	19.09	0.83	0.94	16.30	18.11	26.28	13.35	13.90	23.11	19.52	18.65
		44	Complement factor B	675651986	XP_008992435	86 kDa	18.10	27.36	9.84	1.51	0.54	17.07	7.92	29.31	34.60	4.96	42.52	13.94	5.74
		45	Beta-2-glycoprotein 1	675660951	XP_002748690	42 kDa	13.78	16.36	20.05	1.19	1.46	13.97	10.19	17.18	14.57	17.87	16.64	18.59	21.52
		46	Apolipoprotein C-III	296216257	XP_002754491	11 kDa	12.55	12.68	15.62	1.01	1.24	9.31	19.24	9.10	7.28	16.88	13.87	11.16	20.08
		47	Mannose-binding protein A-like	675708400	XP_009007903	26 kDa	12.51	13.24	12.67	1.06	1.01	13.97	12.45	11.12	12.75	15.88	11.09	16.73	8.61
		48	Vitamin D-binding protein	675649152	XP_008991447	53 kDa	11.87	9.39	11.06	0.79	0.93	6.21	10.19	19.20	7.28	7.94	12.94	12.09	10.04
		49	Kininogen-1	296224808	XP_002758204	72 kDa	10.94	15.17	13.85	1.39	1.27	12.42	11.32	9.10	12.14	14.89	18.49	17.66	10.04
		50	Histidine-rich glycoprotein	296224806	XP_002758205	59 kDa	6.08	4.14	3.97	0.68	0.65	8.54	5.66	4.04	5.46	6.95	0.00	6.51	1.43
		51	Sex hormone-binding globulin	296201365	XP_002747998	43 kDa	5.82	8.06	3.80	1.39	0.65	6.99	3.40	7.08	6.07	7.94	10.17	1.86	5.74
		52	ATP-binding cassette sub-family C member	296231025	XP_002760976	155 kDa	4.01	4.87	7.56	1.21	1.88	2.33	5.66	4.04	3.03	6.95	4.62	6.51	8.61
		53	Apolipoprotein C-I	296234068	XP_002762274	10 kDa	3.68	6.92	5.66	1.88	1.54	3.10	7.92	0.00	6.68	2.98	11.09	5.58	5.74
		54	Complement component C7	390460021	XP_002745085	93 kDa	2.46	3.72	1.86	1.51	0.76	2.33	0.00	5.05	5.46	1.99	3.70	3.72	0.00
		55	Apolipoprotein A-IV	296216255	XP_002754492	46 kDa	2.40	1.21	3.33	0.51	1.39	0.78	3.40	3.03	3.64	0.00	0.00	0.93	5.74
		56	Complement C5	296190703	XP_002743307	188 kDa	2.20	2.97	1.86	1.35	0.84	1.55	0.00	5.05	6.07	0.99	1.85	3.72	0.00
		57	N-acetylmuramoyl-L-alanine amidase	296233165	XP_002761897	62 kDa	1.27	1.37	0.93	1.08	0.73	1.55	2.26	0.00	1.21	1.99	0.92	1.86	0.00
		58	Doublecortin domain-containing protein 5	390470376	XP_003734280	101 kDa	0.97	0.71	0.93			0.78	1.13	1.01	1.21	0.00	0.92	1.86	0.00
		59	Complement component C8 alpha chain	296208071	XP_002750918	66 kDa	0.78	0.40	0.46			2.33	0.00	0.00	1.21	0.00	0.00	0.93	0.00
		60	Ig kappa chain V-III region CLL	675719786	XP_008978552	26 kDa	0.52	0.40	0.00			1.55	0.00	0.00	1.21	0.00	0.00	0.00	0.00
		61	Alpha-1B-glycoprotein	390479473	XP_002762619	55 kDa	0.52	0.20	0.46			1.55	0.00	0.00	0.61	0.00	0.00	0.93	0.00
		62	Alpha-2-antiplasmin	296201026	XP_002747859	53 kDa	0.34	0.31	0.00			0.00	0.00	1.01	0.00	0.00	0.92	0.00	0.00
		63	Serotransferrin	675739694	XP_008982051	77 kDa	0.34	0.00	0.00			0.00	0.00	1.01	0.00	0.00	0.00	0.00	0.00
		64	Apolipoprotein B-100	675724870	XP_008979419	516 kDa	0.26	0.40	0.46			0.78	0.00	0.00	1.21	0.00	0.00	0.93	0.00
		65	Complement C1r subcomponent	675677702	XP_009001757	80 kDa	0.26	0.31	0.00			0.78	0.00	0.00	0.00	0.00	0.92	0.00	0.00
		66	Protein AMBP	296190640	XP_002743269	39 kDa	0.26	0.00	0.00			0.78	0.00	0.00	0.00	0.00	0.00	0.00	0.00
		67	Serum amyloid A-1 protein	675698878	XP_009006032	13 kDa	0.00	0.62	0.00			0.00	0.00	0.00	0.00	0.00	1.85	0.00	0.00
		68	Beta-2-microglobulin	390468617	XP_002753457	14 kDa	0.00	0.20	0.00			0.00	0.00	0.00	0.61	0.00	0.00	0.00	0.00
		69	Coagulation factor V	675746631	XP_008983320	252 kDa	0.00	0.20	0.00			0.00	0.00	0.00	0.61	0.00	0.00	0.00	0.00

*Supplemental Table 1. Relative amounts of all proteins detected by LC/MS during pregnancy. Category 1: Proteins whose relative amount more than doubled or was undetectable initially but increased to ≥ 1 from the base level during pregnancy. Category 1a: Graph peak at second trimester. Category 1b: Graph peak at third trimester. Category 2: Proteins whose relative amount decreased to less than half or with an initially detectable relative amount that decreased to undetectable levels during pregnancy. Category 2a: Graph minimum at second trimester. Category 2b: Graph minimum at third trimester. Category3: Proteins whose relative amount changed within the range of 0.5 to 2 times during pregnancy. *: UniProtKB No., **: GenBank No.*

Supplemental Table 2

		β-actin	PZP	A2ML1
H4	Whole Placenta	3	2	2
	Chorion	3	2	2
	Decidua	3	2	2
H5	Whole Placenta	3	0	1
	Chorion	3	0	1
	Decidua	3	0	1
H6	Whole Placenta	3	1	2
	Chorion	3	1	1
	Decidua	3	1	1
H7	Whole Placenta	3	0	1
	Chorion	3	1	1
	Decidua	3	0	2
H8	Whole Placenta	3	1	1
	Chorion	3	0	1
	Decidua	3	1	2
H9	Whole Placenta	3	1	1
	Chorion	3	1	1
	Decidua	3	1	1
H10	Whole Placenta	3	2	2
	Chorion	3	1	1
	Decidua	3	2	2

		β-actin	PZP	A2ML1
M4	Whole Placenta	3	0	2
	Chorion	3	0	2
	Decidua	3	0	1
M5	Whole Placenta	3	0	2
	Chorion	3	0	2
	Decidua	3	0	1
M6	Whole Placenta	3	0	1
	Chorion	3	0	2
	Decidua	3	0	1
M7	Whole Placenta	3	0	1
	Chorion	3	0	2
	Decidua	3	0	2

Supplemental Table 2. Tissue-specific expression of PZP and A2ML1 mRNA detected by semi-quantitative RT-PCR. Relative PZP and A2ML1 expression was divided into 4 stages (3,2,1,0).

Supplemental Table 3

A.

Sample name	Pregnant type	Tissue type	Samples					
			Blood				Placenta	
			non-P.	1st	2nd	3rd	Weeks	Delivery type
H1	singleton	Plasma	-	12w2d	20w2d	36w2d	-	-
H2	singleton	Plasma	-	14w3d	20w3d	35w3d	-	-
H3	singleton	Plasma	-	12w1d	21w1d	35w1d	-	-
H4	singleton	placenta	-	-	-	-	37w3d	Scheduled-C/S
H5	singleton	placenta	-	-	-	-	37w4d	Scheduled-C/S
H6	singleton	placenta	-	-	-	-	37w5d	Scheduled-C/S
H7	singleton	placenta	-	-	-	-	37w6d	Scheduled-C/S
H8	singleton	placenta	-	-	-	-	37w1d	Scheduled-C/S
H9	singleton	placenta	-	-	-	-	37w6d	Scheduled-C/S
H10	singleton	placenta	-	-	-	-	37w2d	Scheduled-C/S
H11	non-P.	Plasma	+	-	-	-	-	-
H12	non-P.	Plasma	+	-	-	-	-	-
H13	non-P.	Plasma	+	-	-	-	-	-

B.

Sample name	GFP	Pregnant type	Tissue type	Samples					
				Blood				Placenta	Delivery type
				non-P.	50days	100days	120days		
M1	-	triplet	Plasma	-	*P4580_01	*P4580_02	*P4580_03	-	-
M2	-	twin	Plasma	-	*P4586_01	*P4586_02	*P4586_03	-	-
M3	-	twin	Plasma	-	*P4579_01	*P4579_02	-	-	-
M4	-	singleton	placenta	-	-	-	-	+	Scheduled-C/S
M5	-	singleton	placenta	-	-	-	-	+	Scheduled-C/S
M6	-	no data	placenta	-	-	-	-	+	Scheduled-C/S
M7	-	singleton	placenta	-	-	-	-	+	Scheduled-C/S
M8	+	singleton	placenta	-	-	-	-	+	Scheduled-C/S
M9	+	twin	placenta	-	-	-	-	+	Scheduled-C/S
M10	+	singleton	placenta	-	-	-	-	+	Scheduled-C/S
M11	-	non-P.	Plasma	*N0631	-	-	-	-	-
M12	-	non-P.	Plasma	*N3764	-	-	-	-	-
M13	-	non-P.	Plasma	*N3929	-	-	-	-	-
M14	-	non-P.	Plasma	*N4042	-	-	-	-	-
M15	-	non-P.	Plasma	*N4255	-	-	-	-	-

Supplemental Table 3. Human (A) and marmoset (B) samples.
*Non-P.: Non-pregnant; C/S: Caesarean section. For marmoset, 50, 100, and 120 days correspond to the first, second, and third trimester, respectively. *: sample names conform to Supplemental Fig. 1.*
W: weeks; d: days.

Supplemental Table 4

Primer Name	Sequence
Human-PZP-F	5'-CACAGGAAACCGTCCTGCTT-3'
Human-PZP-R	5'-ACTCAGCAACCACAGACTCAT-3'
Human-A2ML1-F	5'-CTGTCGCTTGAAGCAGAACTC-3'
Human-A2ML1-R	5'-TTCACCCTCTACCACCGTCT-3'
Human-beta actin-F	5'-CCACGGCTGCTTCCAG-3'
Human-beta actin-R	5'-GGGTACATGGTGGTGC-3'
Marmoset-PZP-F	5'-ACTGTGCCCCAAACTTGTGA-3'
marmoset-PZP-R	5'-CTCTCAGCCAGGACGTCAGA-3'
marmoset-A2ML1-F	5'-CCCCTATCAAGTGGCCGAAT-3'
marmoset-A2ML1-R	5'-GGGGTGCCGTAATTTGGAGA-3'
marmoset-beta actin-F	5'-CCACGGCTGCTTCCAG-3'
marmoset-beta actin-R	5'-GGGTACATGGTGGTGC-3'

Supplemental Table 4. List of primer sequences used for RT-PCR analysis in this study.
F: Forward primer; R: reverse primer.
